# Ginseng extract improves pancreatic islet injury and promotes β-cell regeneration in T2DM mice

**DOI:** 10.3389/fphar.2024.1407200

**Published:** 2024-06-25

**Authors:** Jianying Yin, Yuanfeng Huang, Ke Wang, Qin Zhong, Yuan Liu, Zirui Ji, Yiwen Liao, Zhiyuan Ma, Weijian Bei, Weixuan Wang

**Affiliations:** ^1^ Traditional Chinese Medicine Research Institute, Guangdong Pharmaceutical University, Guangzhou, Guangdong, China; ^2^ Guangdong Provincial Research Center of Integration of Traditional Chinese Medicine and Western Medicine in Metabolic Diseases, Guangdong Pharmaceutical University, Guangzhou, Guangdong, China; ^3^ Baishan Institute of Science and Technology, Baishan, Jilin, China

**Keywords:** ginseng extract, type 2 diabetes mellitus, β-cell regeneration, pancreatic islet injury, Akt

## Abstract

**Introduction:**

*Panax ginseng C. A. Mey*. (Araliaceae; Ginseng Radix et Rhizoma), a traditional plant commonly utilized in Eastern Asia, has demonstrated efficacy in treating neuro-damaging diseases and diabetes mellitus. However, its precise roles and mechanism in alleviating type 2 diabetes mellitus (T2DM) need further study. The objective of this study is to explore the pharmacological effects of ginseng extract and elucidate its potential mechanisms in protecting islets and promoting β-cell regeneration.

**Methods:**

The T2DM mouse model was induced through streptozotocin combined with a high-fat diet. Two batches of mice were sacrificed on the 7th and 28th days following ginseng extract administration. Body weight, fasting blood glucose levels, and glucose tolerance were detected. Morphological changes in the pancreatic islets were examined via H & E staining. Levels of serum insulin, glucagon, GLP-1, and inflammatory factors were measured using ELISA. The ability of ginseng extract to promote pancreatic islet β-cell regeneration was evaluated through insulin & PCNA double immunofluorescence staining. Furthermore, the mechanism behind β-cells regeneration was explored through insulin & glucagon double immunofluorescence staining, accompanied by immunohistochemical staining and western blot analyses.

**Results and Discussion:**

The present research revealed that ginseng extract alleviates symptoms of T2DM in mice, including decreased blood glucose levels and improved glucose tolerance. Serum levels of insulin, GLP-1, and IL-10 increased following the administration of ginseng extract, while levels of glucagon, TNF-α, and IL-1β decreased. Ginseng extract preserved normal islet morphology, increased nascent β-cell population, and inhibited inflammatory infiltration within the islets, moreover, it decreased α-cell proportion while increasing β-cell proportion. Mechanistically, ginseng extract might inhibit ARX and MAFB expressions, increase MAFA level to aid in α-cell to β-cell transformation, and activate AKT-FOXM1/cyclin D2 to enhance β-cell proliferation. Our study suggests that ginseng extract may be a promising therapy in treating T2DM, especially in those with islet injury.

## 1 Introduction

As a metabolic disorder, diabetes mellitus (DM) is characterized by the dysfunction of pancreatic islets, leading to chronic hyperglycemia as its primary hallmark. DM encompasses a spectrum of complications and carries an unfavorable prognosis. The 10th edition of the 2021 IDF Global Diabetes Map reveals that a staggering 537 million adults, spanning the age range of 20–79, are currently afflicted with DM globally (Magliano and Boyko, 2021). China is the country with the largest number of diabetic patients in the world (Magliano and Boyko, 2021). From 2011 to 2021, the number of diabetic patients in China has increased from 90 million to 140 million, with an increase of 56% (Magliano and Boyko, 2021). It is expected that in the next 20 years, although the growth rate of diabetes prevalence in China will tend to decline, the total number will increase to 164 million in 2030 and 175 million in 2045 (Magliano and Boyko, 2021). Moreover, the annual rise in the patient count exacerbates its impact on public health and imposes a substantial economic cost (Cole and Florez, 2020). The International Diabetes Federation reports a consistent escalation in global expenditures related to DM, reaching around $966 billion in 2021, and anticipated to further increase to $1054 billion by 2045 (Sun et al., 2022). The etiology and pathogenesis of DM are intricate and multifaceted, presenting a challenging puzzle that remains incompletely unraveled despite extensive investigations. Pancreatic β-cells are universally acknowledged as pivotal in maintaining glucose homeostasis through the secretion of insulin (INS) (Xiao et al., 2018). Emerging research has underscored that a vital pathogenic factor of DM lies in the targeted demolition of β-cells, which comprise a majority of islet cells (65%–80%) (Xiao et al., 2018). It is worth noting that, the percentages of islet cell types may vary among different species. For example, in human pancreatic islet cells, 70% are composed of β-cells, 20% are composed of α-cells, with the remaining <10% being δ-cells and <5% PP cells (Cabrera et al., 2006). In contrast, in rodents, pancreatic islets typically consist of 75%–80% β-cells and 15%–20% α-cells (Walker et al., 2021). The destruction of β-cells precipitates a decrease in functional β-cell mass, a hallmark observed in both type 1 diabetes mellitus and type 2 diabetes (T2DM) (Yi et al., 2020). However, the underlying factors contributing to the functional reduction in β-cell mass are distinctly diverse. In the context of type 1 diabetes, the phenomenon is primarily attributed to the immune system’s assault on β-cells (Beyan et al., 2003; McLaughlin et al., 2016; Eizirik et al., 2020). Conversely, in the case of T2DM, the decrement in β-cell mass is more closely linked to apoptosis/necrosis, stemming from the initial hyperactive response of β-cells in compensating for peripheral insulin resistance (Butler et al., 2003; Eizirik et al., 2020; Chen et al., 2023a). Consequently, the preservation of functional β-cell mass holds paramount significance in the initiation and advancement of DM, as well as in the targeted interventions for T1DM and T2DM (Moin and Butler, 2019).

In DM patients, comprehending the process of regeneration holds paramount importance in harnessing the innate regenerative mechanisms of organs to restore the functional quality of β-cells (Sever and Grapin-Botton, 2020). Dor *et al.* showed that new β-cells could be produced by replication of existing β-cells, not from stem cells (Dor et al., 2004). They hypothesized that the primary method for the adult β-cell population’s physiological upkeep is through the self-replication of current β-cells (Dor et al., 2004). The augmentation of β-cell number and quality can occur through self-replication under normal physiological conditions or following various triggers such as pregnancy, pancreatectomy, insulin resistance, and obesity (Lingohr et al., 2002). In addition, it is noteworthy that duct cells and acinar cells residing within the pancreas possess the potential to convert into β-cells (Doke et al., 2023; Son and Accili, 2023).

The quantity and quality of β-cells are strictly regulated, with transcription factors V-Maf musculoaponeurotic fibrosarcoma oncogene homolog B (MAFB) and V-Maf musculoaponeurotic fibrosarcoma oncogene homolog A (MAFA) serving as pivotal players in the later stages of β-cell differentiation (Xiafukaiti et al., 2019). In α-cells, silencing MAFB results in a delay in the maturation of β-cells (Xiafukaiti et al., 2019), whereas MAFA implicates in the replication/survival of postnatal β-cells and the preservation of β-cell function (Nishimura et al., 2006). Additionally, the activation of AKT pathway is essential for the quality and functionality of β-cells (Buteau et al., 2001). During β-cell replication, cells re-enter the cell cycle, which is governed by cell cycle proteins that control the G1 phase (Xiafukaiti et al., 2019). AKT exercises control over the cell cycle process by modulating the levels and subcellular distribution of proteins involved in cell cycle regulation (Fatrai et al., 2006; Mao et al., 2022). Furthermore, chronic inflammation severely impairs insulin secretion and sensitivity (Zhou et al., 2024). Extensive research underscores those various pathogenic mechanisms, such as lipotoxicity, glucotoxicity, and endoplasmic reticulum stress, can initiate inflammatory responses and lead to dysfunction of pancreatic islet β-cells (Nordmann et al., 2017). Multiple inflammatory cytokines can control the quantity and function of β-cells. Pro-inflammatory cytokines, such as IL-6, IL-1β, and TNF-α, can diminish the expressions of important transcription factors MAFA and MAFB, whereas IL-10, an anti-inflammatory cytokine, inhibits the synthesis of IL-6, IL-1β, and TNF-α (van Exel et al., 2002). In mice, IL-10 injection augmented β-cell mass, the proportion of β-cells, and levels of serum insulin, highlighting the importance of IL-10 in protecting islet β-cells as well as enhancing insulin secretion (Hong et al., 2021).

According to traditional Chinese medicine, DM is referred to as Xiaoke, the pathogenesis of diabetes mellitus mainly lies in the deficiency of yin fluid and the excess of dryness-heat, with yin deficiency as the root cause and dryness-heat as the superficial manifestation. DM has been treated with traditional botanical drugs for a lengthy period, owing to their distinctive advantages that stem from their multi-component, multi-target nature and the reduced occurrence of side effects (Chen et al., 2023a; Chan et al., 2023). Due to their notable effectiveness, researchers are increasingly investigating the potential of traditional botanical drugs in treating DM. The use of *P. ginseng* C.A. Meyer (Araliaceae; Ginseng Radix et Rhizoma) (*Panax ginseng*) in traditional Chinese medicine can be traced back to about 5,000 years ago (Yun, 2001). In traditional Chinese medicine theory, *P. ginseng* is believed to play a role in revitalizing life energy by fully nourishing the qi of the spleen, lung, heart, and kidney. Meanwhile, qi promotes the production of blood. *Panax ginseng* enables the human body to produce blood, restore qi and blood, and then nourish the mind through qi supplementation, thus calming the mind (Jin et al., 2019). *Panax ginseng* has been used in traditional medicine to treat various diseases, such as cardiovascular disease and DM (Hao and Xiao, 2019; Li et al., 2019; Pan et al., 2020). The Huanglian Decoction, recorded in the Treatise on Febrile Diseases, is composed of *P. ginseng*, Coptis chinensis Franch. (Ranunculaceae), Zingiber acuminatum Valeton (Zingiberaceae), Glycyrrhiza uralensis Fisch (Leguminosae), *etc.*, can improve yin deficiency and, therefore has a positive effect on the treatment of DM (Pan et al., 2020). ShengMai-Yin, a famous prescription first recorded in Qian Jin Yao Fang, is composed of Radix ginseng, Radix Ophiopogonis (Ophiopogon japonicas Ker-Gawl., Liliaceae) and Fructus Schisandrae (Schisandra chinensis Baill., Magnoliaceae), and can be used to treat DM, palpitations, shortness of breath, *etc.* (Li et al., 2019). As a wonderful botanical drug, *P. ginseng* has been used in preventing and treating numerous diseases in modern medicine. The dried root and rhizome of *P. ginseng* have gained remarkable popularity on a global scale over the past 3 decades (Basha and Saumya, 2013; Jung et al., 2021). Recent investigations have spotlighted the efficacy of *P. ginseng* in effectively alleviating neurodegenerative diseases like stroke, Parkinson’s disease, senile dementia, and other neuro-damaging conditions, attributed to its neuroregenerative properties (Li et al., 2020). Furthermore, *P. ginseng* has exhibited protective effects in DM and other metabolic diseases (Basha and Saumya, 2013; Jung et al., 2021). Research indicates that oral administration of *P. ginseng* reduces blood glucose levels and improves glucose metabolism in STZ-induced diabetic rats (Abdelazim et al., 2019). In *db/db* mice, 8 weeks of ginseng extract treatment can reduce body weight, fasting blood glucose, and HbA1c levels (Jeon et al., 2013). Mechanistically, the blood sugar level-lowering effect of *P. ginseng* in T2DM mice could potentially be attributed to its modulation of the expression of the glucose transporter protein GLUT (Jeon et al., 2013). In addition, research indicates that *P. ginseng* can promote the restoration of immune homeostasis in T1DM mice (Hong et al., 2012). More importantly, various clinical studies have shown that *P. ginseng* exhibited anti-DM effects (Chen et al., 2019). Patients recently diagnosed with non-insulin-dependent DM were treated with *P. ginseng* for 8 weeks and saw a reduction in fasting plasma glucose levels and an improvement in glycosylated hemoglobin activity (Sotaniemi et al., 1995). Another study showed that participants with impaired fasting glucose levels were found to benefit from an 8-week administration of hydrolyzed *P. ginseng* extract, with reduced fasting and postprandial blood glucose levels (Park et al., 2014). Moreover, 4-week *P. ginseng* treatment decreased the insulin resistance index in patients with T2DM (Ma et al., 2008). Numerous pharmacological studies have shown that *P. ginseng* exerts a lipid-lowering effect in patients with T2DM (Vuksan et al., 2019; Jovanovski et al., 2021). Additionally, the levels of inflammatory factors, IL-6 and TNF-α, are significantly reduced after *P. ginseng* treatment (Mohammadi et al., 2019). It can be seen that previous studies on *P. ginseng* for T2DM mainly focused on regulating glucose metabolism, reducing lipid levels, and anti-inflammation. As β-cell plays a crucial role in DM, enhancing β-cell self-replication capabilities and promoting β-cell regeneration hold significant importance for the treatment of DM (Benthuysen et al., 2016; Kerper et al., 2022). However, whether *P. ginseng* plays a role in promoting islet β-cell regeneration remains unclear, with the underlying mechanism yet to be elucidated. Currently, there are scarcely any reported investigations in this area. Therefore, the novelty and significance of our research lies in exploring the mechanisms of ginseng extract in promoting the regeneration of pancreatic β-cells in diabetic mice. The completion of our research holds significant importance towards achieving rapid and effective treatment and even cure for DM and provides valuable guidance for the development of new drugs.

The main objective of the present research was to explore the protective and regenerative effects of ginseng extract on pancreatic β-cells within an *in vivo* context. Our findings indicate that ginseng extract may promote β-cell regeneration, proliferation, and the conversion of α-cells into β-cells, potentially offering treatment for DM resulting from insufficient pancreatic β-cells. Therefore, this research may offer novel insights for DM prevention and treatment strategies as well as provide a scientific foundation for the utilization of ginseng extract in addressing DM and other metabolic disorders.

## 2 Materials and methods

### 2.1 Preparation of the ginseng extract

Ginseng extract (batch number, S230101; date of production, 1 January 2023) was provided by Hongjiu Biotech Co., Ltd. (Jiling, China). Ginseng extract was procured from the dried roots and rhizome of *P. ginseng*, sourced from five-year-old plants cultivated in Changbai Mountain, Jilin Province. The test reports of the ginseng extract are shown in Supplementary Figures S1, S2. The flow chart of the extraction process of ginseng extract is shown in Supplementary Figure S3. The extraction process involved several sequential steps: comminuting the ginseng into coarse powder, extracting ginsenosides with hot water, and adding alkali (CaO) to eliminate impurities. Subsequently, the extract’s pH was adjusted to neutral with acid solution. To further refine the extract, it was subjected to a macrocellular resin column. The pigment was removed by washing with water until colorless, followed by an additional wash with 70% ammonia alcohol until it achieved colorlessness. The ginsenosides were dissolved in ethanol and eluted, with subsequent recovery of ethanol under reduced pressure, yielding the total ginsenosides.

The quantification of total ginsenoside content was conducted through high-performance liquid chromatography (HPLC). The chromatographic conditions were provided in the Supplementary Material S1. HPLC revealed that the total ginsenoside content is 30.5%. Among the constituents, ginsenoside Rb1 accounted for 13.73%, followed by ginsenoside Rd at 4.83%, ginsenoside Rg1 at 5.51%, and ginsenoside Re at 6.43% (Supplementary Figure S4). The HPLC fingerprint of ginseng extract is shown in Supplementary Figure S5. The HPLC fingerprint of ginsenoside Rg1, Re, Rb1, and Rd is shown in Supplementary Figure S6. The quality standard and extraction protocol were referenced from the Chinese Pharmacopoeia (2020 edition; page 408–409, item: TOTAL GINSENOSIDE GINSENG ROOT) (Supplementary Figure S7).

### 2.2 Animals and treatment strategy

We obtained male C57BL/6 mice aged 7 weeks from ZhuHai Bestest Bio-Tech Co., Ltd. (Zhuhai, China). During the experiment, mice were kept in cages that were specific pathogen-free. The housing environment was kept at a controlled temperature of 22°C ± 3°C with a light/dark cycle of 12 h each. Both water and food were provided *ad libitum* to the mice. Guangdong Pharmaceutical University’s Animal Ethics Committee evaluated and authorized the animal experiments (Approval No. gdpulac2020080).

After a week of acclimatization, the control group was given a regular diet, whereas the remaining groups received a high-fat diet (HFD). The methodology employed for establishing the T2DM mouse model drew upon the experimental approach outlined by Yang *et al.* (Yang et al., 2022). Briefly, following a 4-week period of HFD consumption, mice were injected intraperitoneally with 40 mg/kg streptozotocin (STZ) (dissolved in 0.1 M citric acid-sodium citrate buffer, pH 4.5) for 5 days to establish the T2DM mouse model. Concurrently, the control group was kept on a regular diet and received intraperitoneal injections of the same amount of citrate buffer. The fasting blood glucose (FBG) was assessed 2 days following the last STZ injection. FBG levels ≥11.1 mmol/L suggested that the T2DM mice model had been successfully established.

On the initial day of STZ injection, concomitant preventive drug administration was initiated. Six groups of mice were randomly assigned (n = 10 per group): control group, T2DM model group, GE-L group (T2DM mice treated with 60 mg/kg ginseng extract), GE-M group (T2DM mice treated with 120 mg/kg ginseng extract), GE-H group (T2DM mice treated with 240 mg/kg ginseng extract), and MET group (T2DM mice treated with 350 mg/kg metformin) (Inoue et al., 2021). A 0.5% solution of carboxymethylcellulose sodium (CMC-Na) was used to dissolve all drugs administered. Ginseng extract or metformin was administered daily via gavage each morning. Both the control and T2DM model mice orally received an equivalent amount of the vehicle (0.5% CMC-Na). Following the start of drug administration, the mice were split into two separate groups and euthanized after 7 and 28 days of treatment, as shown in the animal testing diagram (Supplementary Figure S8).

### 2.3 Oral glucose tolerance test (OGTT)

Following a 6-h fast, glucose (2 g/kg) was given by gavage (Luo et al., 2021; Mao et al., 2022). Blood glucose levels were assessed at designated intervals following the intake of glucose, including at 0, 30, 60, and 120 min. The measurements were carried out utilizing a glucose meter. The Area Under the Curve (AUC) was computed to measure the results of OGTT.

### 2.4 Serum biochemical indices analyses

After anesthesia with tribromoethanol (0.01 mL/g), blood samples were obtained via the orbital vein extraction technique using capillary tubes, then euthanasia was performed by cervical dislocation after a 12 h fasting period. After being collected, these blood samples were kept at room temperature in enzyme-free tubes for 1 h. Subsequently, the samples were subjected to centrifugation for 20 min at 4°C and 3500 r/min, collecting the supernatants. Levels of insulin, glucagon, IL-1β, TNF-α, IL-10, and GLP-1 were quantified following the recommendations outlined by Meimian (Jiangsu, China).

### 2.5 Hematoxylin-eosin (H & E), immunohistochemical, and immunofluorescent staining

The techniques for H&E, immunohistochemical, and immunofluorescent staining were performed in accordance with the methods described previously (Hsiao et al., 2019; Wei et al., 2020). After the mice were sacrificed, the pancreatic tissues were collected and fixed in 4% paraformaldehyde before being embedded in paraffin. Following that, 4 µm thick sections were prepared and stained. Sections were subjected to hematoxylin and eosin staining for histological analysis, and pancreatitis scores were assessed utilizing the 0–4 scoring system as previously described (Schmidt et al., 1992). To prepare for immunohistochemical staining, 5% BSA was used to block any non-specific binding sites for 1 h. Following this, the sections were left to incubate overnight with an anti-ARX antibody (diluted at a ratio of 1:200; Abcam, Cambridge, UK). Sections were rinsed thrice with PBS before being treated for 1 h with goat anti-rabbit secondary antibody. Following another round of washing, the sections were exposed to diaminobenzidine solution prior to microscopic examination. Immunofluorescence staining involved labeling sections with anti-PCNA antibody (1:1000; Abcam), then applying anti-insulin antibody (1:200, Abcam), or a combination of anti-insulin (1:200) and anti-glucagon (1:400) antibodies (Proteintech, California, United States).

A microscope (BX53; Olympus, Tokyo, Japan) was used to examine the slides. We manually counted the amount of newborn islet β-cells (INS^+^PCNA^+^). The calculation of islet cells was carried out using ImageJ software (National Institutes of Health, Bethesda, MD, United States). The ratio of α- and β-cells was counted using the Image-Pro Plus software (Media Cybernetics, Silver Spring, MD, United States).

### 2.6 Western blotting analysis

The Western blotting procedure adhered to the methodology outlined by Chen et al., 2023b. RIPA buffer with protease and phosphatase inhibitors (Beyotime, Shanghai, China) was used to extract protein samples from pancreatic tissue. A BCA protein quantification kit (Beyotime) was used to determine protein concentration. After being separated by a 10% SDS-PAGE gel, protein samples were subsequently moved to a PVDF membrane (Millipore, Boston, United States). The membranes were obstructed using 5% nonfat milk for 1 h at room temperature, followed by an overnight incubation at 4°C with a primary antibody. This study utilized primary antibodies (diluted at a ratio of 1:1000) against GAPDH (Cell Signaling Technology, Danvers, MA, United States), FOXM1 (Abcam), cyclin D2 (Abcam), AKT (Cell Signaling Technology), P-AKT (Cell Signaling Technology), MAFA (Cell Signaling Technology), and MAFB (Santa Cruz, CA, United States). Following the primary antibody incubation, the membrane was treated with either HRP-conjugated anti-rabbit or anti-mouse secondary antibody at room temperature for 1 h (1:1000 dilution) (Cell Signaling Technology). Subsequently, the membrane was rinsed using TBST, following incubation with ECL solution (Yeasen, Shanghai, China). Protein bands were identified with a Bio-Rad chemiluminescence imager (Hercules, United States), following calculation with Image Lab 6.0 software (Bio-Rad).

### 2.7 Statistical analyses

All statistical analyses were performed using GraphPad Prism 8 (GraphPad Software Inc., La Jolla, United States). The statistical evaluations were performed by either one-way ANOVA or two-way ANOVA to ascertain the significance of variations among different groups. A *p*-value less than 0.05 was considered to have statistical significance.

## 3 Results

### 3.1 Ginseng extract reduces FBG levels

**FIGURE 1:**
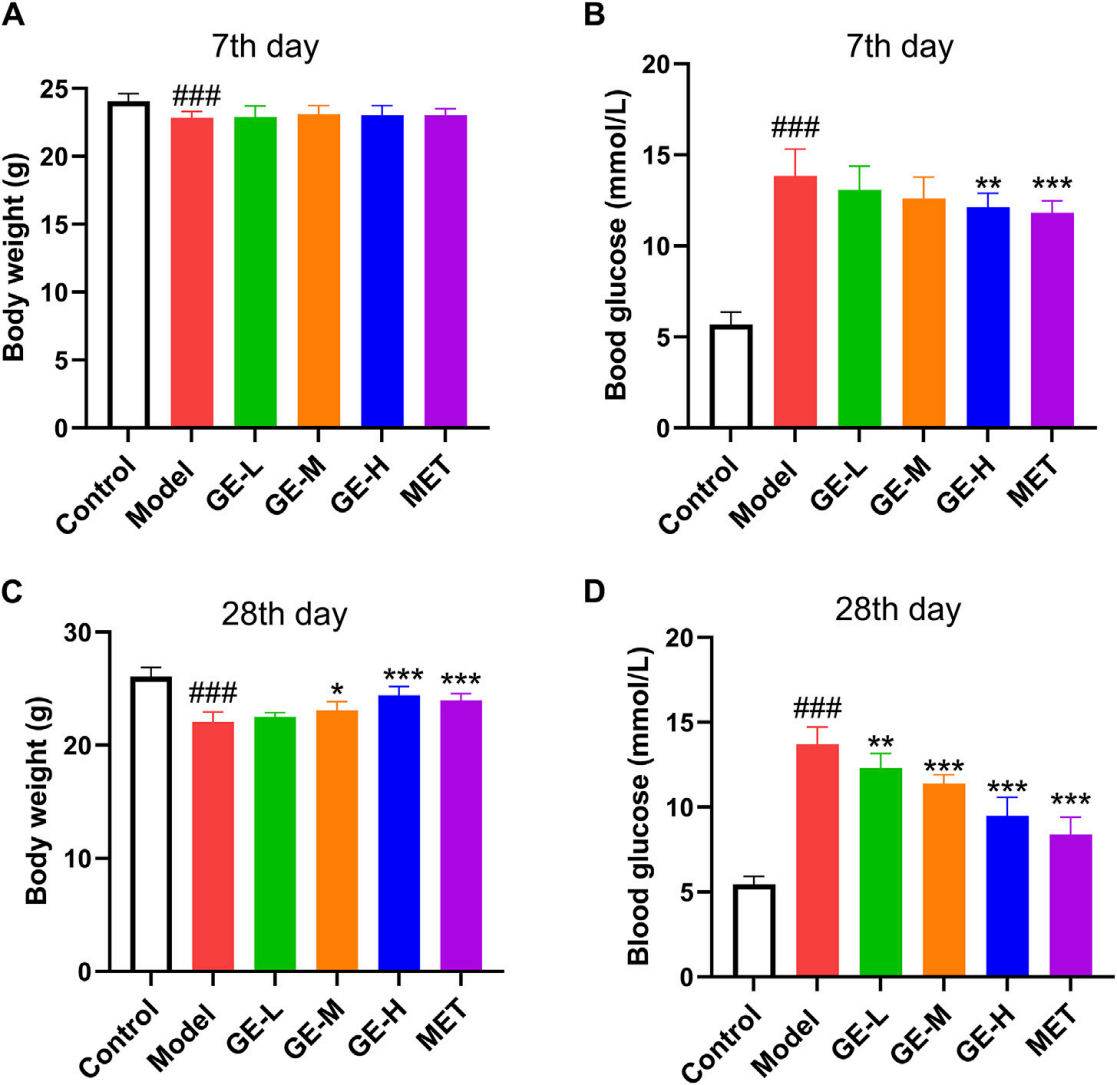
Ginseng extract reduces blood glucose levels in type 2 diabetes mellitus (T2DM) mice. **(A)** Body mass after 7 days of drug treatment. **(B)** Blood sugar levels after 7 days of taking the medication. **(C)** Body mass after 28 days of drug treatment. **(D)** Blood sugar levels after 28 days of taking the medication. Data are presented as mean ± SEM, *n* = 8–10. ^###^ *p* < 0.001 (vs. control group); ^*^ *p* < 0.05, ^**^ *p* < 0.01, ^***^ *p* < 0.001 (vs. model group). Low-dose of ginseng extract, GE-L; medium-dose of ginseng extract, GE-M; high-dose of ginseng extract, GE-H; metformin, MET.

Compared with the control group, mice that received STZ injections exhibited a slight reduction in body weight and a notable rise in blood glucose levels (*p* < 0.001), as depicted in Figures 1A–D. After 7 days of medication, in comparison to the model mice, no marked alterations were observed in terms of body weight (Figure 1A). Whereas, mice treated with high-dose ginseng extract and metformin both showed a marked decrease in FBG, as shown in Figure 1B (*p* < 0.01). As illustrated in Figures 1C, D, after a 28-day administration period, when contrasted with the model mice, the medium- and high-dose ginseng extract-treated groups and metformin-treated group exhibited an enhancement in body weight (*p* < 0.05), along with a marked reduction in FBG levels (*p* < 0.001). These results suggest that ginseng extract holds promise for effectively alleviating T2DM symptoms in mice.

### 3.2 Ginseng extract improved pancreatic islet function of T2DM mice

**FIGURE 2:**
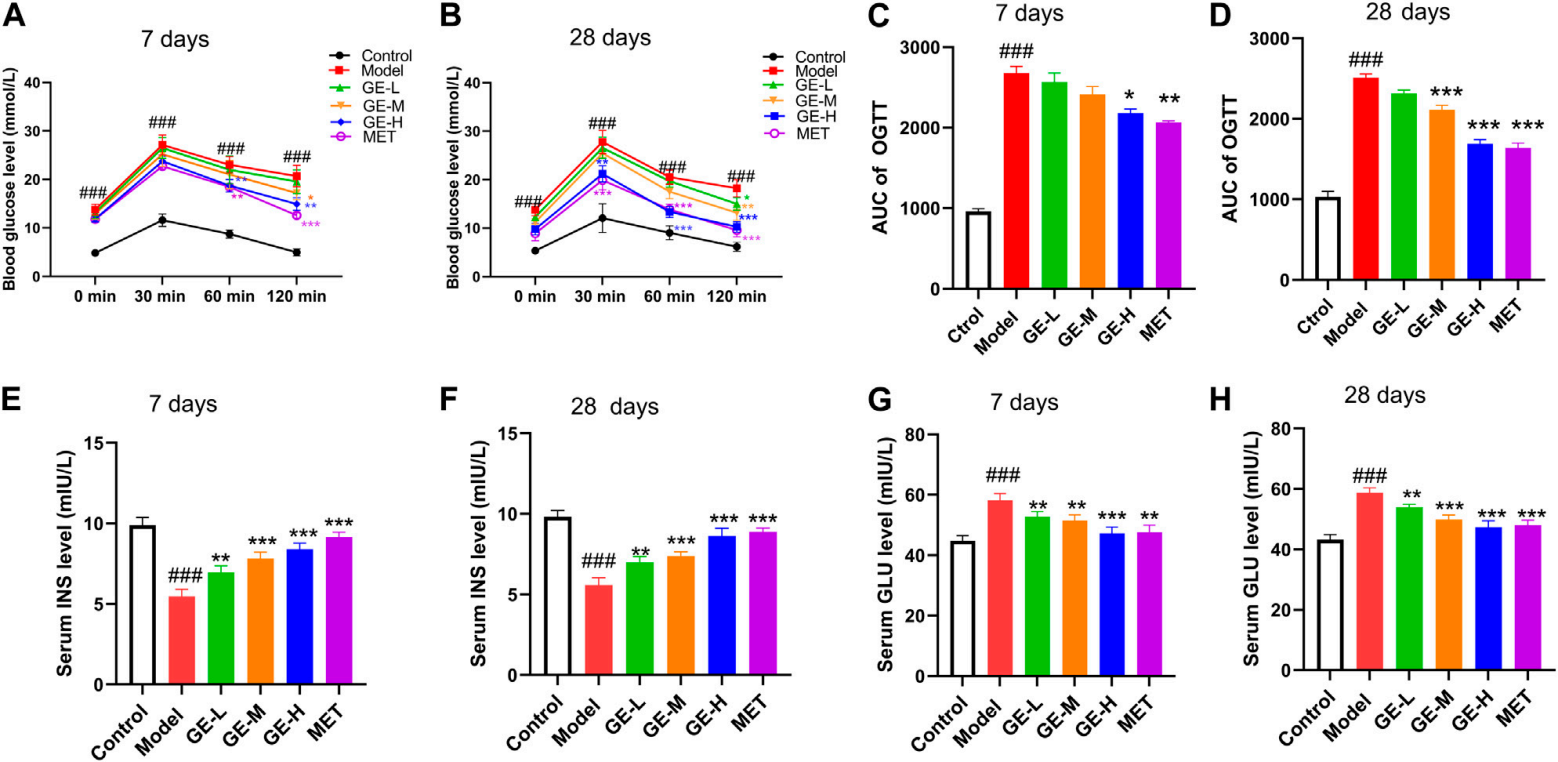
Impacts of ginseng extract on glucose tolerance and insulin resistance in mice with T2DM. **(A)** Impact of ginseng extract on glucose tolerance after 7-day drug administration. **(B)** Impact of ginseng extract on glucose tolerance after 28-day drug administration. **(C)** The area under the curve (AUC) in **(A)**. **(D)** The area under the curve (AUC) in **(B)**. **(E)** Impact of ginseng extract on serum insulin (INS) levels after 7-day drug administration. **(F)** Impact of ginseng extract on INS levels after 28-day drug administration. **(G)** Impact of ginseng extract on serum glucagon (GLU) after 7-day drug administration. **(H)** Impact of ginseng extract on serum GLU after 28-day drug administration. Data are presented as mean ± SEM, n = 6–7. ^###^ *p* < 0.001 (vs. control group); ^*^ *p* < 0.05, ^**^ *p* < 0.01, ^***^ *p* < 0.001 (vs. model group).

As depicted in Figures 2A–D, the blood sugar levels of the mice reached their highest point 30 min after receiving glucose. It is noteworthy that the FBG in the control group gradually decreased to its original level after reaching a peak. Conversely, the model group of mice sustained high FBG even after the peak, resulting in a notable enhancement in the area under the glucose tolerance curve (AUC) (Figures 2A–D). These observations suggest that mice in the model group exhibited pronounced insulin resistance and severe impairment of pancreatic islet function. Although the low-dose ginseng extract-treated group exhibited no substantial improvement in OGTT outcomes following 7 days or 28 days of ginseng extract administration, the middle-dose and high-dose ginseng extract-treated groups showcased a decrease in AUC compared to the model mice (Figures 2C, D). Similar results were observed in the metformin-treated group, which displayed a notable decrease in AUC when compared with the model mice (Figures 2C, D) (*p* < 0.001). These results suggest that both ginseng extract and metformin possess the capacity to improve impaired glucose tolerance of T2DM mice.

In comparison to the control mice, the model mice exhibited a marked decrease in serum insulin levels as well as a substantial rise in serum glucagon levels (Figures 2E–H) (*p* < 0.001). When compared with the model mice, all treatment groups exhibited enhanced serum insulin levels, concomitant with decreased glucagon levels (Figures 2E–H). These outcomes collectively demonstrate that ginseng extract could promote insulin secretion, suppress glucagon secretion, improve pancreatic islet function, and ameliorate insulin resistance in T2DM mice.

### 3.3 Ginseng extract increased serum GLP-1 levels and inhibited inflammatory responses in T2DM mice

**FIGURE 3:**
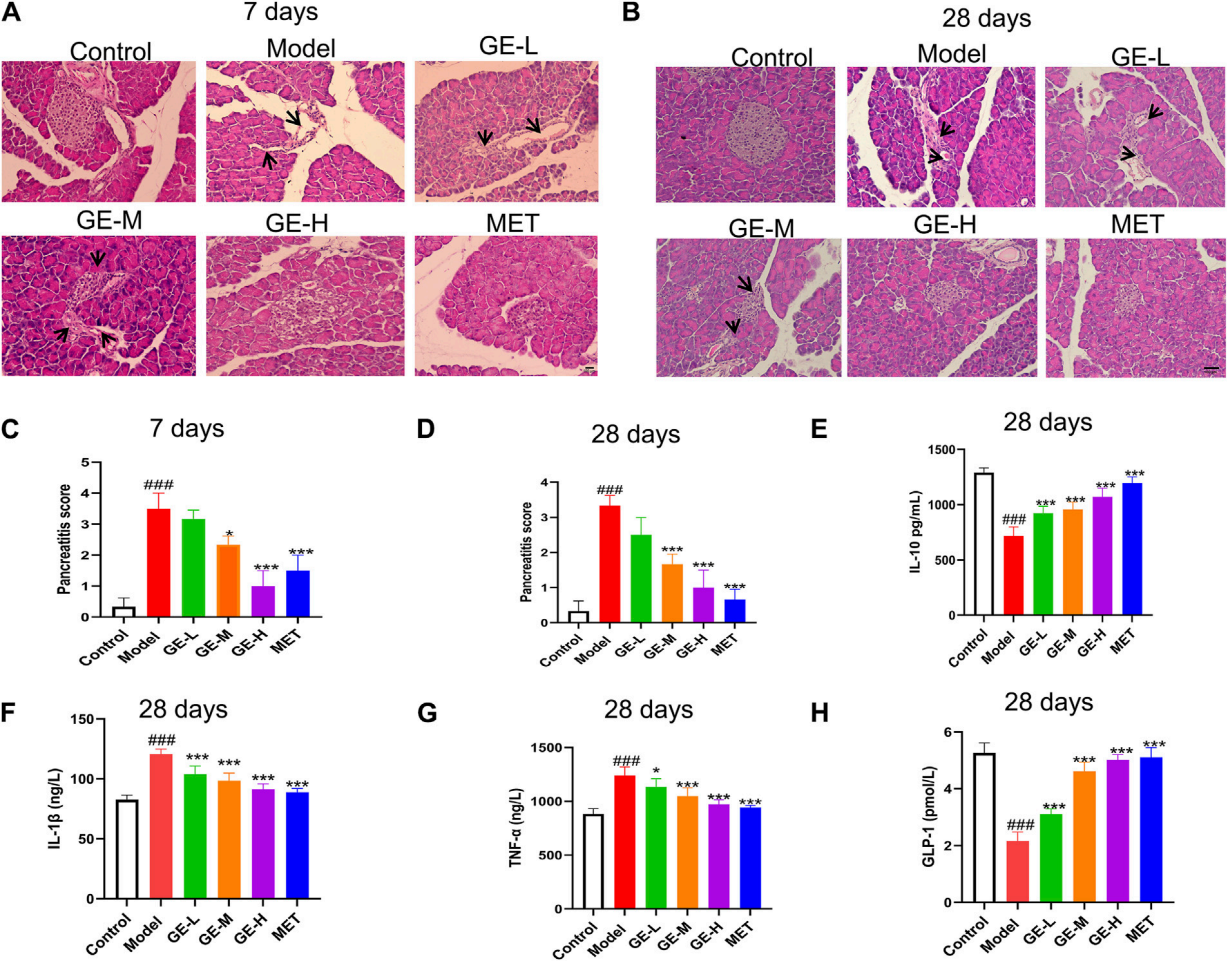
Impacts of ginseng extract on pancreatic tissue inflammation and serum inflammatory factors in mice with T2DM. **(A)** The pancreas was stained with Hematoxylin-eosin (H & E) after 7 days of treatment with ginseng extract or metformin (Scale bar = 100 μm). Inflammatory infiltrating cells are shown by black arrows. **(B)** Pancreatic tissues were stained with H & E after 28 days of drug treatment (Scale bar = 100 μm). Inflammatory infiltrating cells are shown by black arrows. **(C, D)** Pancreatitis scoring in different groups of mice administered ginseng extract or metformin for 7 or 28 days, *n* = 3. **(E)** Serum IL-10 levels were measured after 28-day drug treatment. **(F)** Serum IL-1β levels were measured after 28-day drug treatment. **(G)** Serum TNF-α levels were measured after 28 days of drug treatment. **(H)** Serum GLP-1 levels were measured after 28 days of drug treatment. **(E**–**H)**, n = 7-8. Data are presented as mean ± SEM. ^###^ *p* < 0.001 (vs. control group); *p < 0.05, ***p < 0.001 (vs. model group).

Pancreatic tissue was evaluated for structural pathological changes using H and E staining. As indicated in Figures 3A, B, control mice showed normal structural characteristics, including clear boundaries and no obvious infiltration of inflammatory cells. Conversely, within the model group, there was a pronounced atrophy of pancreatic islets, severe morphological disruption, and irregular structure. Furthermore, the boundaries of these pancreatic islets were indistinct, accompanied by the emergence of numerous vacuoles and severe infiltration of inflammatory cells. Following treatment with ginseng extract, the morphology of the pancreatic islet exhibited a return to regularity, with visible boundaries. Notably, the inflammatory cell infiltration observed in the model group was significantly alleviated. These results suggest that ginseng extract confers a degree of protection to pancreatic islets and inhibits inflammatory cell infiltration. These findings align with the pancreatitis scoring results, as depicted in Figures 3C, D.

Apoptosis and β-cell dysfunction are known to be induced by cellular inflammatory cytokine stress (Ortis et al., 2010). ELISA kits were employed to measure the serum levels of specific inflammatory cytokines after 28 days of treatment. Results showed a notable reduction in serum IL-10 levels (*p* < 0.001) in mice from the model group, with increased IL-1β and TNF-α levels when compared with the control mice (*p* < 0.001) (Figures 3E–G). After receiving ginseng extract and metformin, the IL-1β and TNF-α levels were noticeably reduced when compared with the model mice (*p* < 0.05), while the IL-10 level exhibited a marked increase (*p* < 0.001) (Figures 3E–G). Furthermore, the serum levels of GLP-1 were assessed, revealing a decline in GLP-1 levels within the model group, while administration of ginseng extract or metformin for 28 days resulted in a notable enhancement of GLP-1 levels (*p* < 0.001) (Figure 3H). However, we are uncertain if the changes in insulin and glucagon levels are due to the shift in GLP-1 levels. This may be a concomitant phenomenon, or it might indicate a causal relationship and further research is needed. These findings collectively indicate that ginseng extract inhibits inflammatory response, promotes GLP-1 secretion, and protects β-cells against inflammatory damage in T2DM mice.

### 3.4 Ginseng extract promoted the neogenesis of pancreatic β-cells in T2DM mice

**FIGURE 4:**
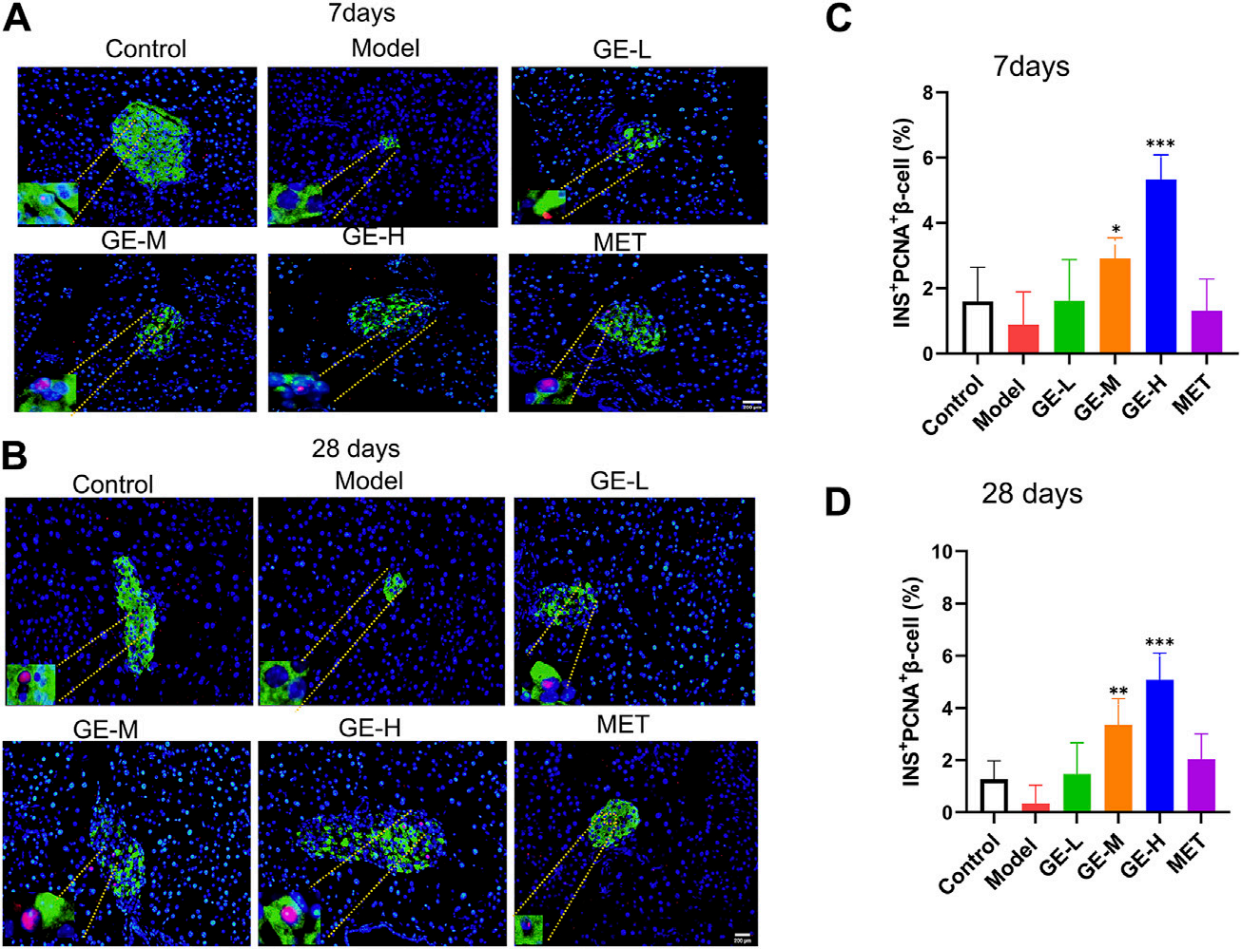
The promotion effect of ginseng extract on islet β-cell neogenesis in T2DM mice. **(A)** Pancreatic PCNA (red) and INS (green) dual immunofluorescence staining in the 7-day batch. **(B)** Pancreatic PCNA (red) and INS (green) dual immunofluorescence staining in the 28-day batch. The blue color represents the DAPI-labeled cell nucleus. Scale bar = 200 μm. **(C, D)** Quantification of islet INS^+^PCNA^+^ β-cells in mice treated for either 7 or 28 days. Data are presented as mean ± SEM, n = 4. *p < 0.05, **p < 0.01, ***p < 0.001 (vs. model group).

Immunofluorescence double staining with PCNA and INS, which represents the neogenesis of β-cells, was performed to assess the β-cell proliferation rate. As illustrated in Figures 4A, B, the islets from both batches of mice in the model group were all destroyed, as evidenced by reduced islet area, diminished insulin secretion, and scarcely observed neogenesis of islet β-cells. Following the administration of ginseng extract, discernible improvements were noted in the pancreatic islet morphology, indicating ginseng extract prevents β-cell destruction. Furthermore, the area size of pancreatic islets increased, and noticeable β-cell neogenesis was observed. Importantly, ginseng extract stimulated insulin secretion and the high-dose ginseng extract treatment group showed better effect. The metformin treatment produced similar results to the low-dose ginseng extract treatment. Specifically, in comparison to the model mice, the metformin-treated group exhibited a gradual recovery in pancreatic islet morphology, a significant increase in area size, and a relatively limited presence of β-cell neogenesis. Consistent with the double staining results, quantification of the INS^+^PCNA^+^ β-cells revealed a decrease in the model mice compared to the control mice, while treatment with medium or high doses of ginseng extract markedly enhanced PCNA^+^INS^+^ β-cell number (Figures 4C, D). These results suggest that ginseng extract can promote the regeneration of pancreatic β-cells in T2DM mice.

### 3.5 Ginseng extract affects the structure and α-/β-cell ratio of pancreatic islets

**FIGURE 5:**
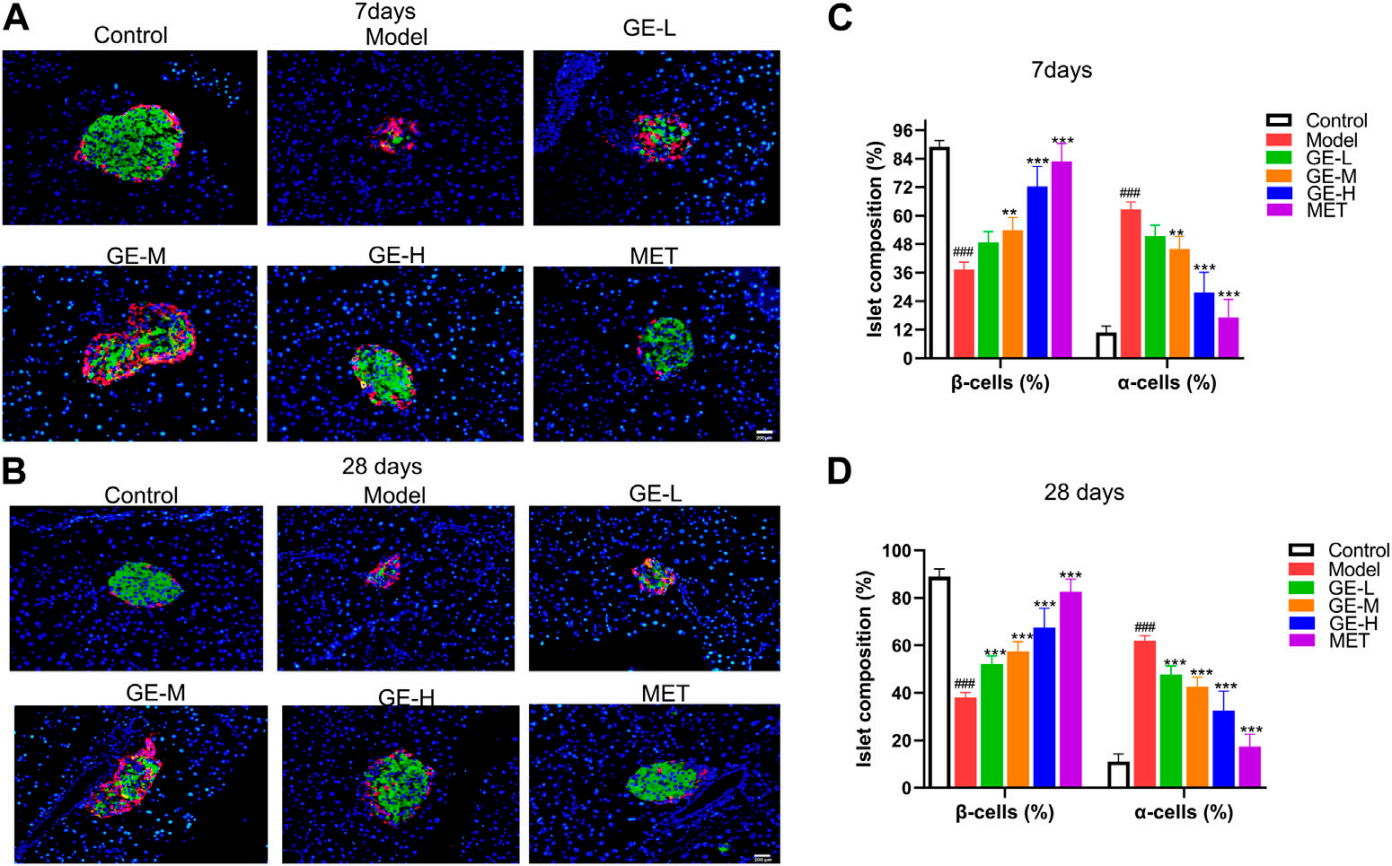
Impact of ginseng extract on islet structure and α-and β-cell ratio. **(A)** Glucagon (red) and insulin (green) dual immunofluorescence staining in the pancreatic tissue sections of T2DM mice after 7 days of administration. **(B)** Glucagon (red) and insulin (green) dual immunofluorescence staining in the pancreatic tissue sections of T2DM mice after 28 days of administration. The blue color represents the DAPI-labeled cell nucleus. Scale bar = 200 μm. **(C, D)** Quantification of islet α-cells and β-cells in mice treated for either 7 or 28 days. Data are presented as mean ± SEM, n = 4. ^###^ *p* < 0.001 (vs. control group); **p < 0.01, ***p < 0.001 (vs. model group).

To investigate alterations in the pancreatic islet structure and changes in α- and β-cell ratio within the islets, pancreatic sections were subjected to dual immunofluorescence staining for insulin and glucagon. As depicted in Figures 5A, B, normal islets predominantly consist of a substantial population of β-cells with a smaller quantity of α-cells. Additionally, in the control mice, islet β-cells are generally found in the central region of the pancreatic islet, while α-cells are located on the outer periphery of the islet, surrounding the β-cells. By comparison, in the model group, serious damage to the pancreatic islet structure led to a significant reduction of β-cell proportion and an enhancement of α-cell proportion (Figures 5A–D). After being treated with ginseng extract, a gradual restoration of islet structure was observed, along with a rise in the number of β-cells and a reduction of α-cells (Figures 5A–D). Additionally, in mice treated with ginseng extract, we observed the emergence of a small group of yellow insulin^+^glucagon^+^ cells, commonly considered transitional cells, during the transformation from α-cells to β-cells (Zhang et al., 2019) (Figures 5A, B). Comparative analysis of the quantitative outcomes from the two batches of mice revealed that the effect between the 7-day administration and 28-day administration showed a slight difference (Figures 5C, D). This may be due to the stimulatory effects of ginseng extract on promoting β-cell proliferation and transformation occur primarily in the early stages, reaching a considerable degree when administered for 7 days. Subsequently, at later stages (administered for 28 days), the primary role of ginseng extract is to protect the newly generated and transformed β-cells and maintain their normal functions, such as insulin secretion. Overall, these studies indicate that ginseng extract may convert α-cells to β-cells as well as preserve β-cells.

### 3.6 Ginseng extract reduced the expression level of ARX

**FIGURE 6:**
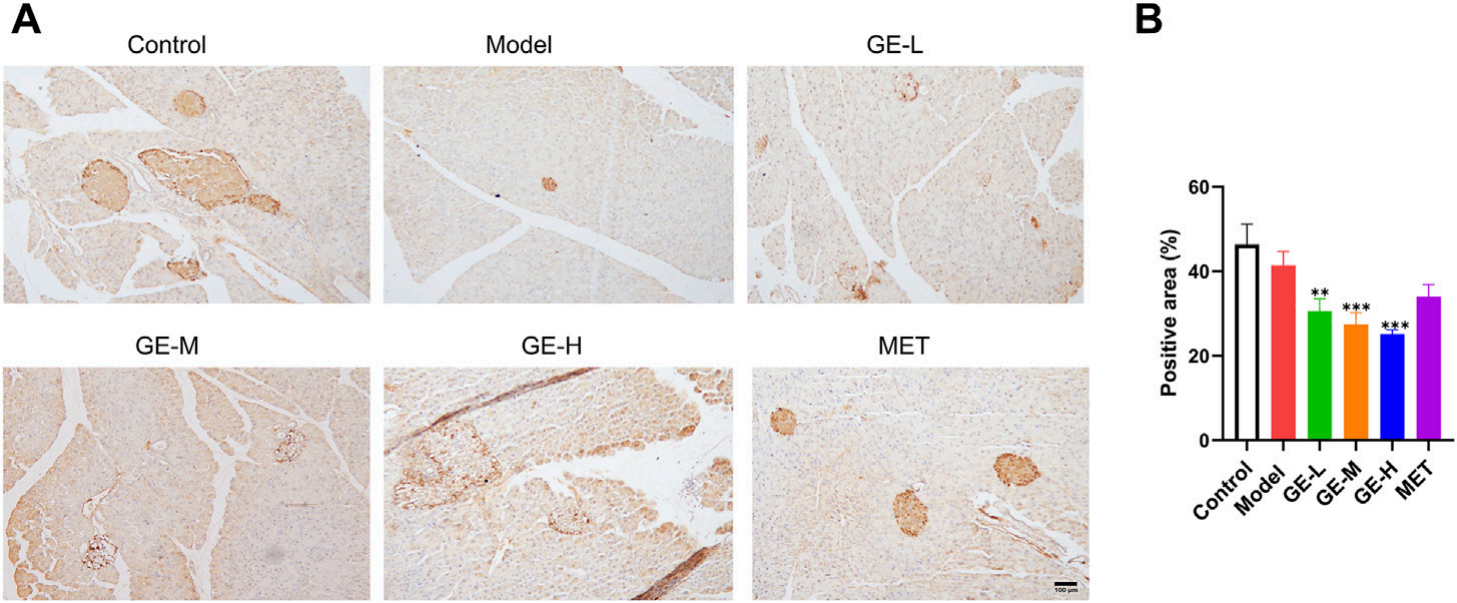
Impact of ginseng extract on ARX level. **(A)** Immunohistochemical staining of pancreatic ARX after 7-day treatment. Scale bar = 100 μm. **(B)** Quantification of ARX expression in mice treated for 7 days. Data are presented as mean ± SEM, n = 3. **p < 0.01, ***p < 0.001 (vs. model group).

Aristaless-related homeobox (ARX) is an important transcription factor that serves as a biomarker for pancreatic α-cells (Xu and Xu, 2019). Mice in the 7-day treatment group had significantly increased β-cell proportion and decreased α-cell proportion, therefore, the 7-day batch was used to conduct immunohistochemical staining for ARX. Despite the noticeable reduction in the size of pancreatic islets within the model group, the quantitative assessment of ARX-positive signal intensity did not exhibit a substantial difference compared with the control mice (Figures 6A, B). Furthermore, despite the significant enlargement of pancreatic islets after receiving ginseng extract, the ARX expression was greatly reduced when compared with the model mice, particularly in the high-dose ginseng extract-treated group (Figures 6A, B). These results indicate that ginseng extract might act by inhibiting ARX expression to facilitate the transformation of α-cells to β-cells.

### 3.7 Ginseng extract enhanced the level of MAFA while reducing the level of MAFB

**FIGURE 7:**
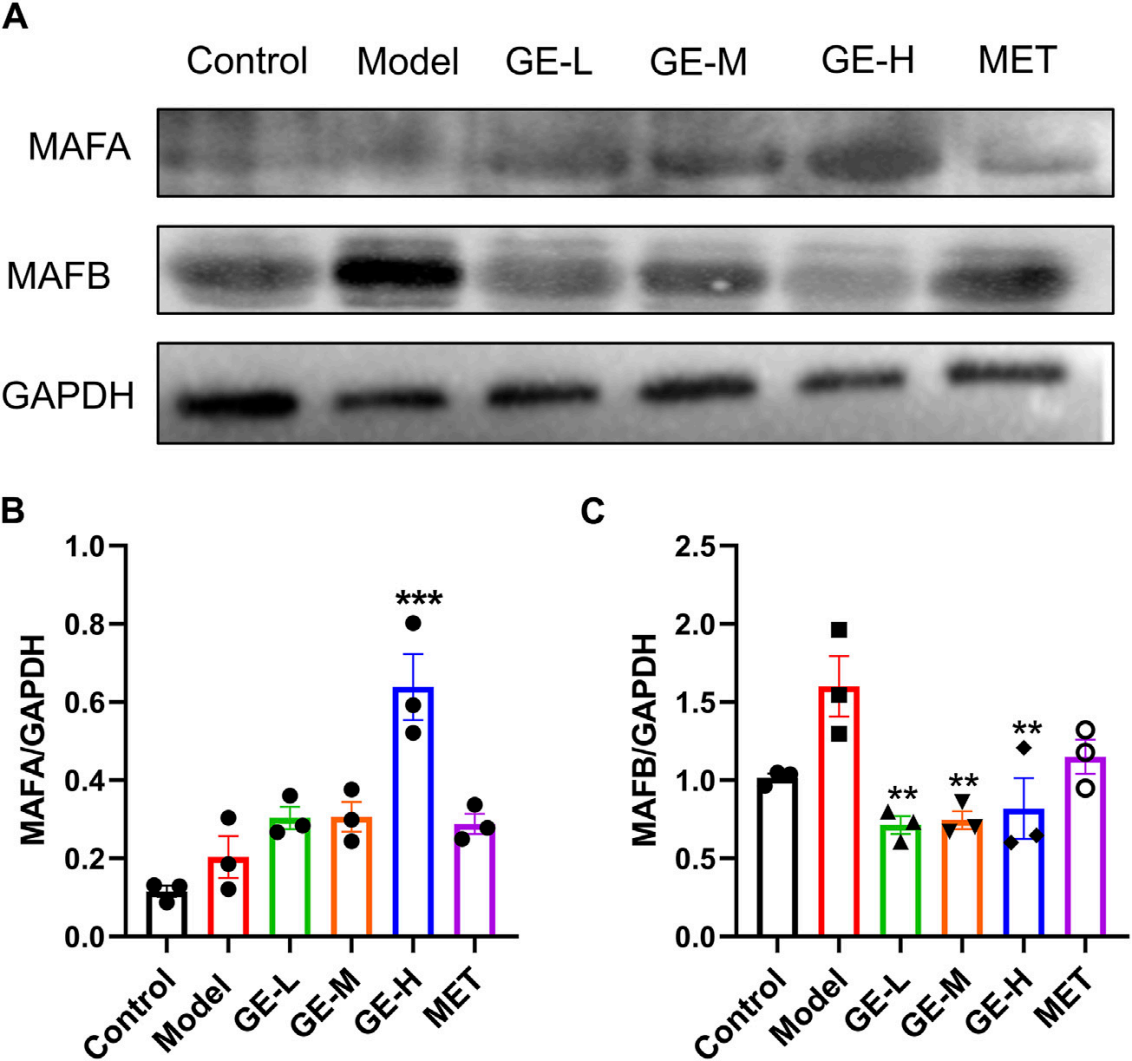
Impact of ginseng extract on MAFA and MAFB levels. **(A)** Pancreatic MAFA and MAFB levels in the 7-day batch. **(B)** The quantitative result of MAFA/GAPDH. **(C)** The quantitative result of MAFB/GAPDH. Data are presented as mean ± SEM, n = 3. **p < 0.01, ***p < 0.001 (vs. model group).

MAFB is specifically expressed in maturing α-cells, whereas MAFA serves as a marker for mature β-cells (Nishimura et al., 2006). The pancreatic protein levels of MAFA and MAFB from the 7-day batch were measured. Compared with the control mice, MAFA levels were slightly elevated in the model mice, however, the observed discrepancy failed to attain statistical significance (Figures 7A, B). Additionally, compared to the model mice, all ginseng extract-treated groups, particularly the high-dose group, and the metformin-treated group exhibited increased expression levels of MAFA (Figures 7A, B). The MAFB levels exhibited a modest increase in the model mice in comparison to the control mice, but without a significant difference (Figures 7A, C). All ginseng extract-treated mice showed decreased MAFB expressions in comparison to the model mice. These findings suggest that ginseng extract might facilitate the transformation from α-cells to β-cells by regulating MAFA and MAFB expression levels.

### 3.8 Ginseng extract activated AKT and upregulated the expressions of FOXM1 and cyclin D2

**FIGURE 8:**
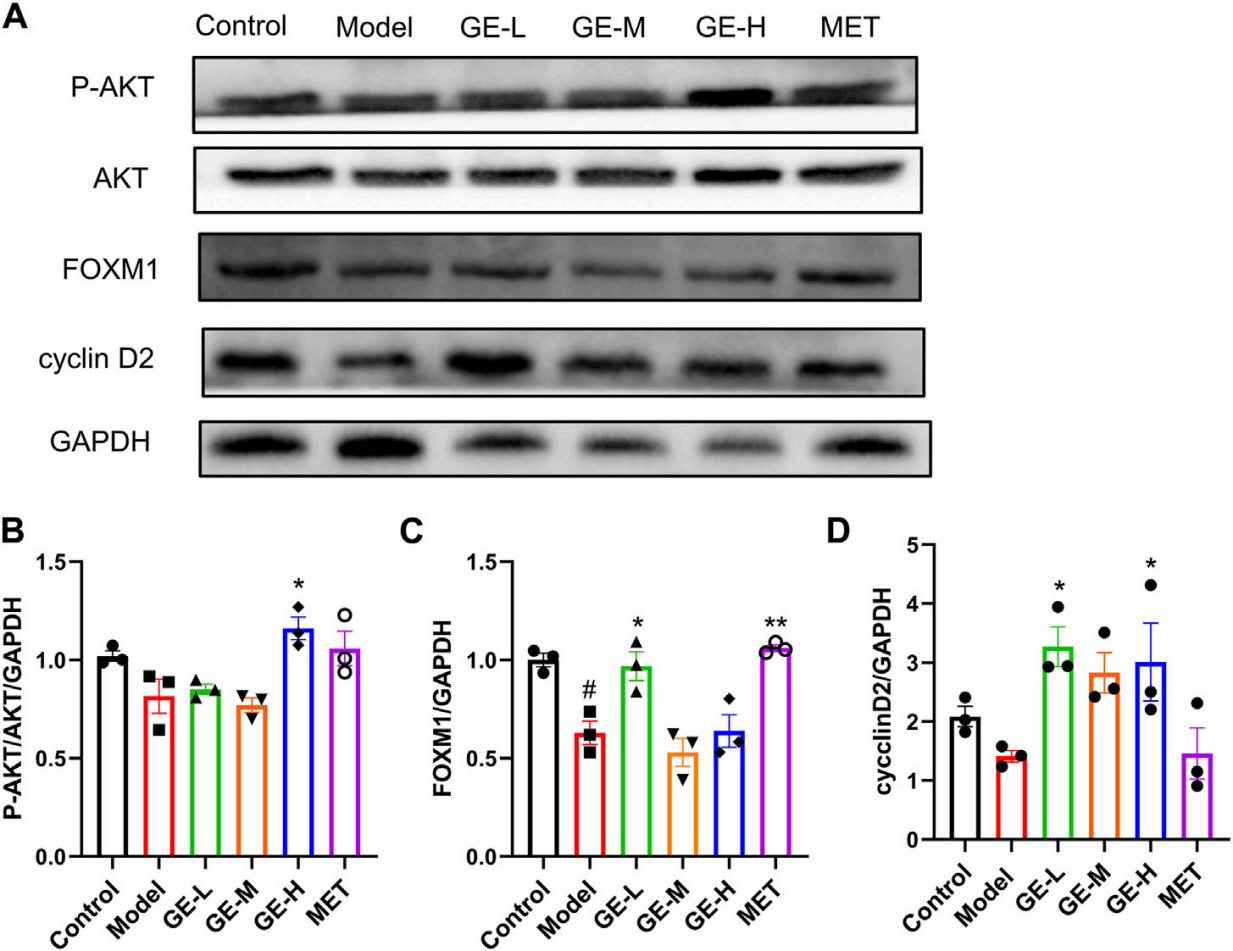
Impact of ginseng extract on the AKT signaling cascade. **(A)** Pancreatic P-AKT, AKT, FOXM1, and cyclin D2 levels in the 7-day batch. **(B)** The quantitative result of P-AKT/AKT/GAPDH. **(C)** The quantitative result of FOXM1/GAPDH. **(D)** The quantitative result of cyclinD2/GAPDH. Data are presented as mean ± SEM, n = 3. #*p* < 0.05 (vs. control group); *p < 0.05, **p < 0.01 (vs. model group).

Pancreatic AKT, FOXM1, and cyclin D2 levels in the 7-day batch were examined through Western blot analysis, which demonstrated that in the model mice, P-AKT/AKT, FOXM1, and cyclin D2 levels were slightly decreased in comparison to the control mice. Following intervention with ginseng extract, the expressions of these proteins showed an upward trend (Figures 8A–D). Upon administration of metformin, the P-AKT/AKT, and FOXM1 levels were increased compared to the model mice (Figures 8A–C). These findings suggest that ginseng extract might enhance the proliferation of islet β-cells through modulation of the AKT pathway.

## 4 Discussion

DM remains a global problem that has not been completely solved. Current treatments primarily target symptoms, aiming at controlling pathological manifestations. Recent studies have demonstrated that compounds extracted from plants have the potential to treat DM. Shikonin possesses anti-diabetic potential with minimal side effects, bringing new hope for the treatment of DM (Saeed et al., 2021). Additionally, (−)-epigallocatechin gallate significantly reduces blood glucose, lipid levels, and oxidative stress in diabetic mice, exhibiting protective effects on liver and kidney functions, indicating its potential as a promising candidate for DM treatment (Soussi et al., 2022). However, until now, there is no way to thoroughly cure DM (Ebrahim et al., 2022). β-cell exerts pivotal effects in maintaining glucose homeostasis through producing biologically active insulin (Xiao et al., 2018). Inadequate functional β-cells are a shared characteristic in different types of DM (Lee et al., 2020; Sever and Grapin-Botton, 2020). Consequently, there is a significant focus on methods to boost the number of β-cells and improve insulin release by either promoting β-cell proliferation or triggering differentiation from alternative cell sources (Ackermann et al., 2018). Regenerating pancreatic β-cells can be achieved through two approaches. The first approach aims to prevent β-cell reduction through the suppression of β-cell necrosis, apoptosis, and dedifferentiation. The alternative method focuses on enhancing the neogenesis of β-cells, including promoting cell proliferation, triggering the transformation from different cell sources, activating pancreatic endocrine progenitor cells, or facilitating the differentiation into β-cells for the purpose of allowing β-cells to undergo endogenous regeneration (Wang et al., 2021). The present study assessed the effectiveness of ginseng extract in stimulating the regeneration of islet β-cells and safeguarding pancreatic islets. The results of our study offer experimental support for using ginseng extract in clinical settings and present a novel approach for managing T2DM.

Traditionally, *P. ginseng* has been associated with anti-aging effects. Research showed that Renshenshouwu extract containing *P. ginseng* extract can stimulate neuro-regeneration by increasing the population of newborn neurons and other related neural cells (Li et al., 2020). This knowledge prompted us to investigate the potential of ginseng extract to promote the regeneration of β-cells. Our results revealed that ginseng extract effectively promoted pancreatic β-cell regeneration, improved the function of the pancreas, enhanced insulin secretion, alleviated insulin resistance, and decreased blood glucose levels. Additionally, we found that ginseng extract had anti-inflammatory effects, protecting pancreatic islets, reducing β-cell loss caused by inflammatory stimuli, and stimulating the transformation from islet α-cells to β-cells.

Our current study illustrated that the administration of ginseng extract increased β-cell ratio and decreased α-cell ratio in T2DM mice. Furthermore, ginseng extract administration resulted in a significant inhibition of ARX expression in T2DM mice. Furthermore, the administration of ginseng extract to mice with T2DM resulted in elevated levels of MAFA and reduced levels of MAFB. ARX, a transcription factor, is crucial in controlling the conversion from α-cells to β-cells. Specifically deactivating ARX and Dnmt1 can successfully prompt α-cell to transform into insulin-expressing cells similar to native β-cell (Chakravarthy et al., 2017). Consistently, specific knockdown of ARX leads to a significant decrease in α-cell and increased quantities of β-cell and δ-cell (Hancock et al., 2010). MAFA serves as a marker for mature β-cells, while MAFB is present in α-cells and developing β-cells (Nishimura et al., 2006). As β-cell mature, MAFB is ultimately replaced by MAFA, leading to the exclusive expression of MAFB in mature α-cells. Research has revealed that as α-cells transform into β-cells, the amount of MAFB decreases over time, while the expression of MAFA increases (van der Meulen and Huising, 2015). Hence, we hypothesized that in T2DM mice, ginseng extract suppressed ARX and MAFB levels while enhancing MAFA expression to promote the transformation from α-cells to β-cells.

The AKT is essential for controlling a variety of proteins that regulate the cell cycle, including FOXM1, cyclin D2, cyclin D1, and p21 (Tschen et al., 2009; Kohata et al., 2022). Our research revealed that ginseng extract activated AKT, and enhanced FOXM1 and cyclin D2 levels in mice with T2DM. Cyclin D2 and FOXM1 have the ability to stimulate G1/S transition by inducing the transcription of multiple cyclins. (Fatrai et al., 2006; Tschen et al., 2017; Kohata et al., 2022). Previous study showed that stimulating the AKT led to higher amounts of cyclin D2, cyclin D1, and p21, leading to a proliferative response in β-cells (Fatrai et al., 2006). Therefore, our findings suggested that ginseng extract might activate the pancreatic AKT-FOXM1/cyclin D2 signaling pathway to promote the proliferation of β-cells.

Due to the increasing incidence of DM, which poses a serious threat to human health (Majety et al., 2023), and the critical role of pancreatic islet β-cell function in regulating diabetic metabolism (Christensen and Gannon, 2019), current research trends focus on exploring ways to treat DM by enhancing β-cell self-replication and promoting β-cell regeneration (Benthuysen et al., 2016; Kerper et al., 2022). Therefore, the identification of drugs with such effects represents an urgent clinical need and our research precisely compensates for the insufficiency in this field.

The current study provides a foundation for understanding the mechanisms underlying islet β-cell regeneration through mice experiments. To expand upon these findings, future research could involve investigating the effects of ginseng extract at the cellular level using mouse or human islet cells. Furthermore, ginseng extract is composed of various ginsenosides, including Rb1, Rd, Rg1, Re, and other ginsenosides, which have been reported to have glucose-regulating effects (Yuan et al., 2012). However, it remains unclear which specific ginsenoside contributes to promoting β-cell regeneration. Further research is needed to elucidate the specific ginsenoside responsible for this effect and uncover the underlying mechanisms, including which signaling pathways and nuclear factors they may target. The completion of the above-mentioned experiments could open up new avenues for the development of T2DM therapies.

## 5 Conclusion

In conclusion, our present results revealed that ginseng extract could alleviate T2DM symptoms, including promoting pancreatic islet β-cell regeneration, improved pancreatic islet injury, and decreased blood glucose levels. These effects appear to be mediated through multiple mechanisms. Firstly, ginseng extract may protect β-cells against inflammatory damage, thereby maintaining the mass and function of β-cells. Additionally, ginseng extract appears to facilitate the transformation from α-cells to β-cells possibly through regulating the pancreatic ARX, MAFA, and MAFB levels, consequently leading to enhanced β-cell number. Moreover, our research suggests that the positive impacts of ginseng extract might be due to stimulated AKT, which leads to accelerated cell cycle progression and increased β-cell proliferation.

## Data Availability

The original contributions presented in the study are included in the article/Supplementary Material, further inquiries can be directed to the corresponding authors.

## Abbreviations

ARX, aristaless-related homeobox; AUC, area under the curve; DAPI, 4′,6-diamidino-2-phenylindole; ECL, enhanced chemiluminescence; ELISA, enzyme-linked immunosorbent assay; FBG, fasting blood glucose; FOXM1, forkhead box M1; GAPDH, glyceraldehyde-3-phosphate dehydrogenase; GLU, glucagon; GLP-1, glucagon-like peptide-1; H and E, hematoxylin-eosin; HFD: high-fat diet; *i. p.*, intraperitoneal; IL-10, interleukin-10; IL-1β, interleukin-1β; INS, insulin; MAFA, V-Maf Musculoaponeurotic fibrosarcoma oncogene Homologue A; MAFB, V-Maf Musculoaponeurotic Fibrosarcoma oncogene Homolog B; MET, metformin; OGTT, oral glucose tolerance test; TNF-ɑ, tumor necrosis factor-ɑ.

## References

[B1] Abdelazim A. Khater S. Ali H. Shalaby S. Afifi M. Saddick S. (2019). Panax ginseng improves glucose metabolism in streptozotocin-induced diabetic rats through 5' adenosine monophosphate kinase up-regulation. Saudi J. Biol. Sci. 26 (7), 1436–1441. 10.1016/j.sjbs.2018.06.001 31762606 PMC6864146

[B2] Ackermann A. M. Moss N. G. Kaestner K. H. (2018). GABA and artesunate do not induce pancreatic α-to-β cell transdifferentiation In Vivo. Cell. Metab. 28 (5), 787–792. 10.1016/j.cmet.2018.07.002 30057067 PMC8129910

[B3] Basha M. P. Saumya S. M. (2013). Influence of fluoride on streptozotocin induced diabetic nephrotoxicity in mice: protective role of Asian ginseng (Panax ginseng) and banaba (Lagerstroemia speciosa) on mitochondrial oxidative stress. Indian J. Med. Res. 137 (2), 370–379. 23563382 PMC3657862

[B4] Benthuysen J. R. Carrano A. C. Sander M. (2016). Advances in β cell replacement and regeneration strategies for treating diabetes. J. Clin. Investig. 126 (10), 3651–3660. 10.1172/jci87439 27694741 PMC5096826

[B5] Beyan H. Buckley L. R. Yousaf N. Londei M. Leslie R. D. (2003). A role for innate immunity in type 1 diabetes? Diabetes Metab. Res. Rev. 19 (2), 89–100. 10.1002/dmrr.341 12673777

[B6] Buteau J. Foisy S. Rhodes C. J. Carpenter L. Biden T. J. Prentki M. (2001). Protein kinase Czeta activation mediates glucagon-like peptide-1-induced pancreatic beta-cell proliferation. Diabetes 50 (10), 2237–2243. 10.2337/diabetes.50.10.2237 11574404

[B7] Butler A. E. Janson J. Bonner-Weir S. Ritzel R. Rizza R. A. Butler P. C. (2003). Beta-cell deficit and increased beta-cell apoptosis in humans with type 2 diabetes. Diabetes 52 (1), 102–110. 10.2337/diabetes.52.1.102 12502499

[B8] Cabrera O. Berman D. M. Kenyon N. S. Ricordi C. Berggren P. O. Caicedo A. (2006). The unique cytoarchitecture of human pancreatic islets has implications for islet cell function. Proc. Natl. Acad. Sci. U. S. A. 103 (7), 2334–2339. 10.1073/pnas.0510790103 16461897 PMC1413730

[B9] Chakravarthy H. Gu X. Enge M. Dai X. Wang Y. Damond N. (2017). Converting adult pancreatic islet α cells into β cells by targeting both Dnmt1 and arx. Cell. Metab. 25 (3), 622–634. 10.1016/j.cmet.2017.01.009 28215845 PMC5358097

[B10] Chan K. W. Kwong A. S. K. Tan K. C. B. Lui S. L. Chan G. C. W. Ip T. P. (2023). Add-on rehmannia-6-based Chinese medicine in type 2 diabetes and ckd: a multicenter randomized controlled trial. Clin. J. Am. Soc. Nephrol. 18 (9), 1163–1174. 10.2215/cjn.0000000000000199 37307005 PMC10564374

[B11] Chen W. Balan P. Popovich D. G. (2019). Review of ginseng anti-diabetic studies. Molecules 24 (24), 4501. 10.3390/molecules24244501 31835292 PMC6943541

[B12] Chen X. Yin J. Zhong Q. Wang K. Zhang X. Liang M. (2023a). Fufang-zhenzhu-tiaozhi formula protects islet against injury and promotes β cell regeneration in diabetic mice. J. Ethnopharmacol. 301, 115791. 10.1016/j.jep.2022.115791 36240976

[B13] Chen Y. K. Liu T. T. Teia F. K. F. Xie M. Z. (2023b). Exploring the underlying mechanisms of obesity and diabetes and the potential of Traditional Chinese Medicine: an overview of the literature. Front. Endocrinol. (Lausanne) 14, 1218880. 10.3389/fendo.2023.1218880 37600709 PMC10433171

[B14] Christensen A. A. Gannon M. (2019). The beta cell in type 2 diabetes. Curr. Diab Rep. 19 (9), 81. 10.1007/s11892-019-1196-4 31399863

[B15] Cole J. B. Florez J. C. (2020). Genetics of diabetes mellitus and diabetes complications. Nat. Rev. Nephrol. 16 (7), 377–390. 10.1038/s41581-020-0278-5 32398868 PMC9639302

[B16] Doke M. Álvarez-Cubela S. Klein D. Altilio I. Schulz J. Mateus Gonçalves L. (2023). Dynamic scRNA-seq of live human pancreatic slices reveals functional endocrine cell neogenesis through an intermediate ducto-acinar stage. Cell. Metab. 35 (11), 1944–1960.e7. 10.1016/j.cmet.2023.10.001 37898119

[B17] Dor Y. Brown J. Martinez O. I. Melton D. A. (2004). Adult pancreatic beta-cells are formed by self-duplication rather than stem-cell differentiation. Nature 429 (6987), 41–46. 10.1038/nature02520 15129273

[B18] Ebrahim N. Shakirova K. Dashinimaev E. (2022). PDX1 is the cornerstone of pancreatic β-cell functions and identity. Front. Mol. Biosci. 9, 1091757. 10.3389/fmolb.2022.1091757 36589234 PMC9798421

[B19] Eizirik D. L. Pasquali L. Cnop M. (2020). Pancreatic β-cells in type 1 and type 2 diabetes mellitus: different pathways to failure. Nat. Rev. Endocrinol. 16 (7), 349–362. 10.1038/s41574-020-0355-7 32398822

[B20] Fatrai S. Elghazi L. Balcazar N. Cras-Méneur C. Krits I. Kiyokawa H. (2006). Akt induces beta-cell proliferation by regulating cyclin D1, cyclin D2, and p21 levels and cyclin-dependent kinase-4 activity. Diabetes 55 (2), 318–325. 10.2337/diabetes.55.02.06.db05-0757 16443763

[B21] Hancock A. S. Du A. Liu J. Miller M. May C. L. (2010). Glucagon deficiency reduces hepatic glucose production and improves glucose tolerance in adult mice. Mol. Endocrinol. 24 (8), 1605–1614. 10.1210/me.2010-0120 20592160 PMC2940466

[B22] Hao D. C. Xiao P. G. (2019). Impact of drug metabolism/pharmacokinetics and their relevance upon traditional medicine-based cardiovascular drug research. Curr. Drug Metab. 20 (7), 556–574. 10.2174/1389200220666190618101526 31237211

[B23] Hong F. Yang Y. Chen B. Li P. Wang G. Jiang Y. (2021). Protein kinase C-θ knockout decreases serum IL-10 levels and inhibits insulin secretion from islet β cells. Islets 13 (1-2), 24–31. 10.1080/19382014.2021.1890963 33719858 PMC8018435

[B24] Hong Y. J. Kim N. Lee K. Hee Sonn C. Eun Lee J. Tae Kim S. (2012). Korean red ginseng (Panax ginseng) ameliorates type 1 diabetes and restores immune cell compartments. J. Ethnopharmacol. 144 (2), 225–233. 10.1016/j.jep.2012.08.009 22925946

[B25] Hsiao C. C. Lin C. C. Hou Y. S. Ko J. Y. Wang C. J. (2019). Low-energy extracorporeal shock wave ameliorates streptozotocin induced diabetes and promotes pancreatic beta cells regeneration in a rat model. Int. J. Mol. Sci. 20 (19), 4934. 10.3390/ijms20194934 31590394 PMC6801760

[B26] Inoue Y. Masuda T. Misumi Y. Ando Y. Ueda M. (2021). Metformin attenuates vascular pathology by increasing expression of insulin-degrading enzyme in a mixed model of cerebral amyloid angiopathy and type 2 diabetes mellitus. Neurosci. Lett. 762, 136136. 10.1016/j.neulet.2021.136136 34311050

[B27] Jeon W. J. Oh J. S. Park M. S. Ji G. E. (2013). Anti-hyperglycemic effect of fermented ginseng in type 2 diabetes mellitus mouse model. Phytother. Res. 27 (2), 166–172. 10.1002/ptr.4706 22511336

[B28] Jin Y. Cui R. Zhao L. Fan J. Li B. (2019). Mechanisms of Panax ginseng action as an antidepressant. Cell. Prolif. 52 (6), e12696. 10.1111/cpr.12696 31599060 PMC6869450

[B29] Jovanovski E. Smircic-Duvnjak L. Komishon A. Au-Yeung F. R. Sievenpiper J. L. Zurbau A. (2021). Effect of coadministration of enriched Korean Red Ginseng (Panax ginseng) and American ginseng (Panax quinquefolius L) on cardiometabolic outcomes in type-2 diabetes: a randomized controlled trial. J. Ginseng Res. 45 (5), 546–554. 10.1016/j.jgr.2019.11.005 34803424 PMC8587487

[B30] Jung E. Pyo M. K. Kim J. (2021). Pectin-lyase-modified ginseng extract and ginsenoside Rd inhibits high glucose-induced ROS production in mesangial cells and prevents renal dysfunction in db/db mice. Molecules 26 (2), 367. 10.3390/molecules26020367 33445772 PMC7828230

[B31] Kerper N. Ashe S. Hebrok M. (2022). Pancreatic β-cell development and regeneration. Cold Spring Harb. Perspect. Biol. 14 (5), a040741. 10.1101/cshperspect.a040741 34580120 PMC9159263

[B32] Kohata M. Imai J. Izumi T. Yamamoto J. Kawana Y. Endo A. (2022). Roles of FoxM1-driven basal β-cell proliferation in maintenance of β-cell mass and glucose tolerance during adulthood. J. Diabetes Investig. 13 (10), 1666–1676. 10.1111/jdi.13846 PMC9533047 35633298

[B33] Lee Y. S. Song G. J. Jun H. S. (2020). Betacellulin-induced α-cell proliferation is mediated by ErbB3 and ErbB4, and may contribute to β-cell regeneration. Front. Cell. Dev. Biol. 8, 605110. 10.3389/fcell.2020.605110 33553143 PMC7859283

[B34] Li S. Qian Y. Xie R. Li Y. Jia Z. Zhang Z. (2019). Exploring the protective effect of ShengMai-Yin and Ganmaidazao decoction combination against type 2 diabetes mellitus with nonalcoholic fatty liver disease by network pharmacology and validation in KKAy mice. J. Ethnopharmacol. 242, 112029. 10.1016/j.jep.2019.112029 31216433

[B35] Li Y. Liang W. Guo C. Chen X. Huang Y. Wang H. (2020). Renshen Shouwu extract enhances neurogenesis and angiogenesis via inhibition of TLR4/NF-κB/NLRP3 signaling pathway following ischemic stroke in rats. J. Ethnopharmacol. 253, 112616. 10.1016/j.jep.2020.112616 32007631

[B36] Lingohr M. K. Buettner R. Rhodes C. J. (2002). Pancreatic beta-cell growth and survival--a role in obesity-linked type 2 diabetes? Trends Mol. Med. 8 (8), 375–384. 10.1016/s1471-4914(02)02377-8 12127723

[B37] Luo Z. Fu C. Li T. Gao Q. Miao D. Xu J. (2021). Hypoglycemic effects of licochalcone A on the streptozotocin-induced diabetic mice and its mechanism study. J. Agric. Food Chem. 69 (8), 2444–2456. 10.1021/acs.jafc.0c07630 33605141

[B38] Ma S. W. Benzie I. F. Chu T. T. Fok B. S. Tomlinson B. Critchley L. A. (2008). Effect of Panax ginseng supplementation on biomarkers of glucose tolerance, antioxidant status and oxidative stress in type 2 diabetic subjects: results of a placebo-controlled human intervention trial. Diabetes Obes. Metab. 10 (11), 1125–1127. 10.1111/j.1463-1326.2008.00858.x 18355331

[B39] Magliano D. J. Boyko E. J. (2021). IDF diabetes atlas *Idf diabetes atlas* . Brussels: International Diabetes Federation. 35914061

[B40] Majety P. Lozada Orquera F. A. Edem D. Hamdy O. (2023). Pharmacological approaches to the prevention of type 2 diabetes mellitus. Front. Endocrinol. (Lausanne) 14, 1118848. 10.3389/fendo.2023.1118848 36967777 PMC10033948

[B41] Mao Y. P. Song Y. M. Pan S. W. Li N. Wang W. X. Feng B. B. (2022). Effect of Codonopsis Radix and Polygonati Rhizoma on the regulation of the IRS1/PI3K/AKT signaling pathway in type 2 diabetic mice. Front. Endocrinol. (Lausanne) 13, 1068555. 10.3389/fendo.2022.1068555 36589810 PMC9794842

[B42] McLaughlin K. A. Richardson C. C. Ravishankar A. Brigatti C. Liberati D. Lampasona V. (2016). Identification of tetraspanin-7 as a target of autoantibodies in type 1 diabetes. Diabetes 65 (6), 1690–1698. 10.2337/db15-1058 26953162

[B43] Mohammadi H. Hadi A. Kord-Varkaneh H. Arab A. Afshari M. Ferguson A. J. R. (2019). Effects of ginseng supplementation on selected markers of inflammation: a systematic review and meta-analysis. Phytother. Res. 33 (8), 1991–2001. 10.1002/ptr.6399 31161680

[B44] Moin A. S. M. Butler A. E. (2019). Alterations in beta cell identity in type 1 and type 2 diabetes. Curr. Diab Rep. 19 (9), 83. 10.1007/s11892-019-1194-6 31401713 PMC6689286

[B45] Nishimura W. Kondo T. Salameh T. El Khattabi I. Dodge R. Bonner-Weir S. (2006). A switch from MafB to MafA expression accompanies differentiation to pancreatic beta-cells. Dev. Biol. 293 (2), 526–539. 10.1016/j.ydbio.2006.02.028 16580660 PMC2390934

[B46] Nordmann T. M. Dror E. Schulze F. Traub S. Berishvili E. Barbieux C. (2017). The role of inflammation in β-cell dedifferentiation. Sci. Rep. 7 (1), 6285. 10.1038/s41598-017-06731-w 28740254 PMC5524956

[B47] Pan L. Li Z. Wang Y. Zhang B. Liu G. Liu J. (2020). Network pharmacology and metabolomics study on the intervention of traditional Chinese medicine Huanglian Decoction in rats with type 2 diabetes mellitus. J. Ethnopharmacol. 258, 112842. 10.1016/j.jep.2020.112842 32333952

[B48] Park S. H. Oh M. R. Choi E. K. Kim M. G. Ha K. C. Lee S. K. (2014). An 8-wk, randomized, double-blind, placebo-controlled clinical trial for the antidiabetic effects of hydrolyzed ginseng extract. J. Ginseng Res. 38 (4), 239–243. 10.1016/j.jgr.2014.05.006 25379002 PMC4213818

[B49] Saeed M. Shoaib A. Tasleem M. Alabdallah N. M. Alam M. J. Asmar Z. E. (2021). Assessment of antidiabetic activity of the shikonin by allosteric inhibition of protein-tyrosine phosphatase 1B (PTP1B) using state of art: an *in silico* and *in vitro* tactics. Molecules 26 (13), 3996. 10.3390/molecules26133996 34208908 PMC8271486

[B50] Schmidt J. Lewandrowski K. Fernandez-del Castillo C. Mandavilli U. Compton C. C. Warshaw A. L. (1992). Histopathologic correlates of serum amylase activity in acute experimental pancreatitis. Dig. Dis. Sci. 37 (9), 1426–1433. 10.1007/bf01296014 1380425

[B51] Sever D. Grapin-Botton A. (2020). Regeneration of the pancreas: proliferation and cellular conversion of surviving cells. Curr. Opin. Genet. Dev. 64, 84–93. 10.1016/j.gde.2020.06.005 32721583

[B52] Son J. Accili D. (2023). Reversing pancreatic β-cell dedifferentiation in the treatment of type 2 diabetes. Exp. Mol. Med. 55 (8), 1652–1658. 10.1038/s12276-023-01043-8 37524865 PMC10474037

[B53] Sotaniemi E. A. Haapakoski E. Rautio A. (1995). Ginseng therapy in non-insulin-dependent diabetic patients. Diabetes Care 18 (10), 1373–1375. 10.2337/diacare.18.10.1373 8721940

[B54] Soussi A. Gargouri M. Magné C. Ben-Nasr H. Kausar M. A. Siddiqui A. J. (2022). (-)-Epigallocatechin gallate (EGCG) pharmacokinetics and molecular interactions towards amelioration of hyperglycemia, hyperlipidemia associated hepatorenal oxidative injury in alloxan induced diabetic mice. Chem. Biol. Interact. 368, 110230. 10.1016/j.cbi.2022.110230 36309138

[B55] Sun H. Saeedi P. Karuranga S. Pinkepank M. Ogurtsova K. Duncan B. B. (2022). IDF Diabetes Atlas: global, regional and country-level diabetes prevalence estimates for 2021 and projections for 2045. Diabetes Res. Clin. Pract. 183, 109119. 10.1016/j.diabres.2021.109119 34879977 PMC11057359

[B56] Tschen S. I. Dhawan S. Gurlo T. Bhushan A. (2009). Age-dependent decline in beta-cell proliferation restricts the capacity of beta-cell regeneration in mice. Diabetes 58 (6), 1312–1320. 10.2337/db08-1651 19228811 PMC2682690

[B57] Tschen S. I. Zeng C. Field L. Dhawan S. Bhushan A. Georgia S. (2017). Cyclin D2 is sufficient to drive β cell self-renewal and regeneration. Cell. Cycle 16 (22), 2183–2191. 10.1080/15384101.2017.1319999 28763258 PMC5736344

[B58] van der Meulen T. Huising M. O. (2015). Role of transcription factors in the transdifferentiation of pancreatic islet cells. J. Mol. Endocrinol. 54 (2), R103–R117. 10.1530/jme-14-0290 25791577 PMC4373662

[B59] van Exel E. Gussekloo J. de Craen A. J. Frölich M. Bootsma-Van Der Wiel A. Westendorp R. G. (2002). Low production capacity of interleukin-10 associates with the metabolic syndrome and type 2 diabetes: the Leiden 85-Plus Study. Diabetes 51 (4), 1088–1092. 10.2337/diabetes.51.4.1088 11916930

[B60] Vuksan V. Xu Z. Z. Jovanovski E. Jenkins A. L. Beljan-Zdravkovic U. Sievenpiper J. L. (2019). Efficacy and safety of American ginseng (Panax quinquefolius L.) extract on glycemic control and cardiovascular risk factors in individuals with type 2 diabetes: a double-blind, randomized, cross-over clinical trial. Eur. J. Nutr. 58 (3), 1237–1245. 10.1007/s00394-018-1642-0 29478187

[B61] Walker J. T. Saunders D. C. Brissova M. Powers A. C. (2021). The human islet: mini-organ with mega-impact. Endocr. Rev. 42 (5), 605–657. 10.1210/endrev/bnab010 33844836 PMC8476939

[B62] Wang K. L. Tao M. Wei T. J. Wei R. (2021). Pancreatic β cell regeneration induced by clinical and preclinical agents. World J. Stem Cells 13 (1), 64–77. 10.4252/wjsc.v13.i1.64 33584980 PMC7859987

[B63] Wei R. Cui X. Feng J. Gu L. Lang S. Wei T. (2020). Dapagliflozin promotes beta cell regeneration by inducing pancreatic endocrine cell phenotype conversion in type 2 diabetic mice. Metabolism 111, 154324. 10.1016/j.metabol.2020.154324 32712220

[B64] Xiafukaiti G. Maimaiti S. Ogata K. Kuno A. Kudo T. Shawki H. H. (2019). MafB is important for pancreatic β-cell maintenance under a MafA-deficient condition. Mol. Cell. Biol. 39 (17), 000800–e119. 10.1128/mcb.00080-19 PMC6692125 31208980

[B65] Xiao X. Guo P. Shiota C. Zhang T. Coudriet G. M. Fischbach S. (2018). Endogenous reprogramming of alpha cells into beta cells, induced by viral gene therapy, reverses autoimmune diabetes. Cell. Stem Cell. 22 (1), 78–90. 10.1016/j.stem.2017.11.020 29304344 PMC5757249

[B66] Xu S. Xu J. P. (2019). Present status and expectation of aristaless-related homeobox (ARX) in endocrine pancreas. Int. J. Dev. Biol. 63 (11-12), 579–587. 10.1387/ijdb.190242sx 32149367

[B67] Yang Y. Q. Tan H. B. Zhang X. Y. Zhang Y. Z. Lin Q. Y. Huang M. Y. (2022). The Chinese medicine Fufang Zhenzhu Tiaozhi capsule protects against renal injury and inflammation in mice with diabetic kidney disease. J. Ethnopharmacol. 292, 115165. 10.1016/j.jep.2022.115165 35247475

[B68] Yi Z. Waseem Ghani M. Ghani H. Jiang W. Waseem Birmani M. Ye L. (2020). Gimmicks of gamma-aminobutyric acid (GABA) in pancreatic β-cell regeneration through transdifferentiation of pancreatic α-to β-cells. Cell. Biol. Int. 44 (4), 926–936. 10.1002/cbin.11302 31903671

[B69] Yuan H. D. Kim J. T. Kim S. H. Chung S. H. (2012). Ginseng and diabetes: the evidences from *in vitro*, animal and human studies. J. Ginseng Res. 36 (1), 27–39. 10.5142/jgr.2012.36.1.27 23717101 PMC3659569

[B70] Yun T. K. (2001). Brief introduction of Panax ginseng C.A. Meyer. J. Korean Med. Sci. 16 (Suppl. l), S3–S5. 10.3346/jkms.2001.16.S.S3 11748372 PMC3202213

[B71] Zhang Z. Hu Y. Xu N. Zhou W. Yang L. Chen R. (2019). A new way for beta cell neogenesis: transdifferentiation from alpha cells induced by glucagon-like peptide 1. J. Diabetes Res. 2019, 2583047. 10.1155/2019/2583047 31001561 PMC6436340

[B72] Zhou Y. Liu K. Tang W. Zhang Y. Sun Y. Wu Y. (2024). β-Cell miRNA-503-5p induced by hypomethylation and inflammation promotes insulin resistance and β-cell decompensation. Diabetes 73 (1), 57–74. 10.2337/db22-1044 37847900

