# Supplementary material for: Ginseng extract improves pancreatic islet injury and promotes β-cell regeneration in T2DM mice
Source: Front Pharmacol. 2024 Jun 25;15:1407200. doi: 10.3389/fphar.2024.1407200 (PMC11234855; doi:10.3389/fphar.2024.1407200)
Supplement: Supplementary file 1 [file DataSheet1.pdf]

宏久生物
HONGJIU BIOTECH

成品检验报告单

报告单编号：B-S230101-CP306

产品名称：人参提取物	植物来源：五加科植物人参（人工种植、四年生或五年生）Panax ginseng C. A. Mey 的干燥根和根茎	
生产批号：S230101	数　量：2kg	
生产日期：2023 年 01 月 02 日	报告日期：2023 年 01 月 08 日	检验依据：企业标准
检验项目	标准规定	检验结果
感官要求		
色泽	本品为浅黄色至棕褐色	符合规定
组织形态	粉末、未有粘结	符合规定
滋、气味	具有人参特有的气味，味甘、微苦，无异味	符合规定
杂质	无正常视力可见外来异物	符合规定
理化指标		
人参总皂苷	≥30.0%HPLC	30.5%
细度(100 目)	≥95%	96.5%
水分	≤8.0%	3.9%
污染物限量		
铅（以 Pb 计）	≤1.49 mg/kg	符合规定
镉（以 Cd 计）	≤1.5 mg/kg	符合规定
总汞（Hg）	≤0.15 mg/kg	符合规定
微生物限量		
菌落总数	≤30000cfu/g	110cfu/g
霉菌和酵母	≤50cfu/g	25cfu/g
大肠菌群	≤0.92MP[illegible]	<0.3MPN/g
沙门氏菌	[illegible]	未检出/25g
金黄色葡萄球菌	0/25g	未检出/25g
结论：符合规定。		
贮藏方法：密闭，置阴凉干燥处	保质期：24 个月	

吉林省宏久生物科技股份有限公司 质检专用章

检验人：　　　　复核人：　　　　质量管理部负责人：

Fig. S1. The examination report of ginseng extract

SGS

Test Report **No: BJFDO071000698FD** **Date: Oct 23 2007**

Client name: JILIN PROVINCE HONGJIU BIOTECH., LTD.
Client address: CHAO YANG TOWN, HUINAN COUNTY, JILIN PROVINCE

The following sample(s) was/were submitted by/ on behalf of the client as:

Sample name: GINSENG EXTRACTED POWDER
Batch: HJ20070901-T
SGS Reference No.: SHFDO071005547FD
SGS Job No: BJFDO071000698FD
Date of receipt: Oct 12 2007
Testing period: Oct 12 2007~ Oct 19 2007

TEST(S) REQUESTED:
Selected test(s) as requested by applicant:
Nutrition test – Calorie, Carbohydrate, Protein, Fat, Sugars, Moisture, Ash

TEST METHOD(S):
Calorie, Carbohydrate: GB 13432-2004 General standard for the labeling of prepackaged foods for special dietary uses
Moisture: GB/T 5009.3-2003 Determination of moisture in foods
Ash: GB/T 5009.4-2003 Determination of ash in foods
Protein: GB/T 5009.5-2003 Determination of protein in foods
Fat: GB/T 5009.6-2003 Determination of fat in foods
Sugars: GB/T 5009.7-2003 Determination of reducing sugar in foods

TEST RESULT(S):

Test items	Test methods	Test results
Moisture	GB/T 5009.3-2003	3.48 g/100g
Ash	GB/T 5009.4-2003	4.04 g/100g
Fat	GB/T 5009.6-2003 I	0.35 g/100g
Protein	GB/T 5009.5-2003	15.6 g/100g
Sugars(as glucose)	GB/T 5009.7-2003	40.5 g/100g

CALCULATE RESULT(S):

Calculate item	Calculate method	Test result
Carbohydrate	GB 13432-2004	76.53 g/100g
Calorie	GB 13432-2004	1579 kJ/100g

Remark: Protein factor is 6.25.

SAMPLE DESCRIPTION: Canary powder in a bag

Page 1 of 1
*** End of Report ***

Signed for and on behalf of SGS

Cathy Yu
Authorized Signature

This Test Report is issued by the Company subject to its General Conditions of Service printed overleaf or attached. Said Conditions are also available upon request or are accessible at www.sgs.com. Attention is drawn to the limitations of liability, indemnification and jurisdictional policies defined therein. The results shown in this Test Report refer only to the sample(s) tested unless otherwise stated and such sample(s) are retained for 30 days only. This Test Report shall not be reproduced except in full, without written approval of the Company.

BJFD 002046

SGS-CSTC Standards Technical Services Co., Ltd. Beijing Branch [illegible]
16/F,Century YuHui Mansion,No.73,Fucheng Road,Beijing,China 100036 t (86-10) 88142851 88145201 f (86-10) 88142852 www.cn.sgs.com
中国・北京・海淀区阜成路73号世纪裕惠大厦16层 邮编:100036 t (86-10) 88142851 88145201 f (86-10) 88142852 e sgs.china@sgs.com

Fig. S2. The test report of the ginseng extract

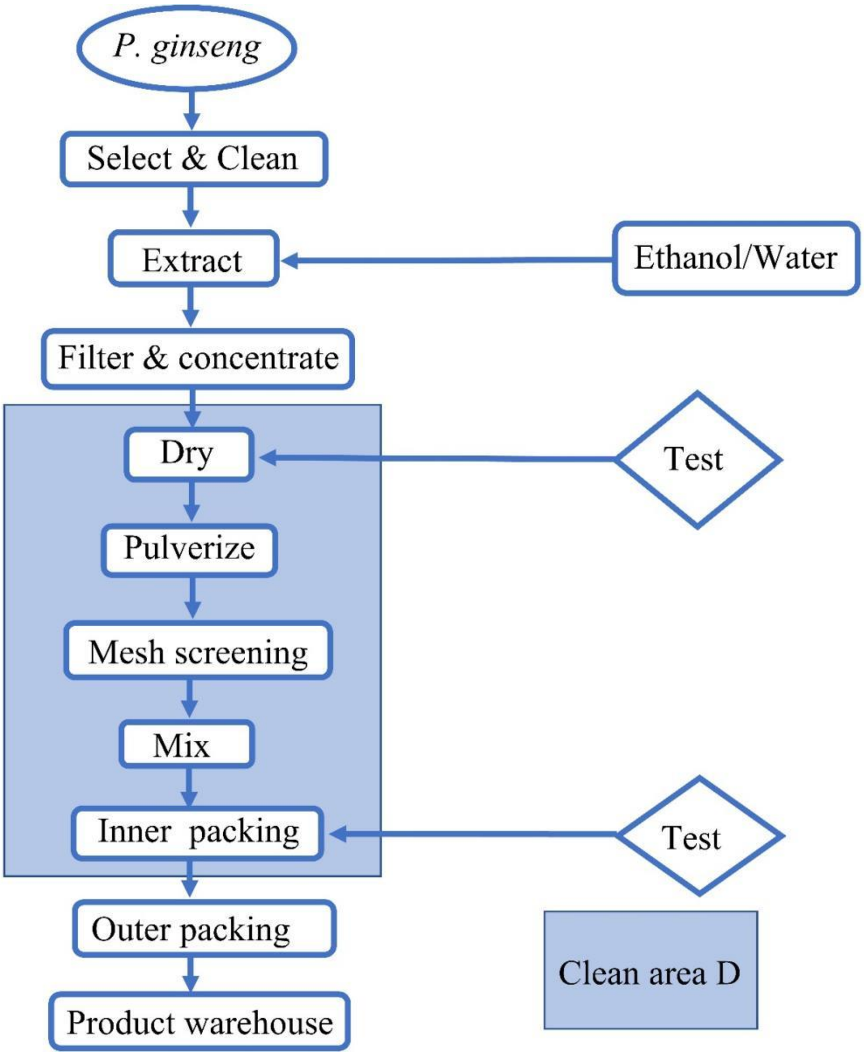

Fig. S3. The flow chart of the extraction process of ginseng extract

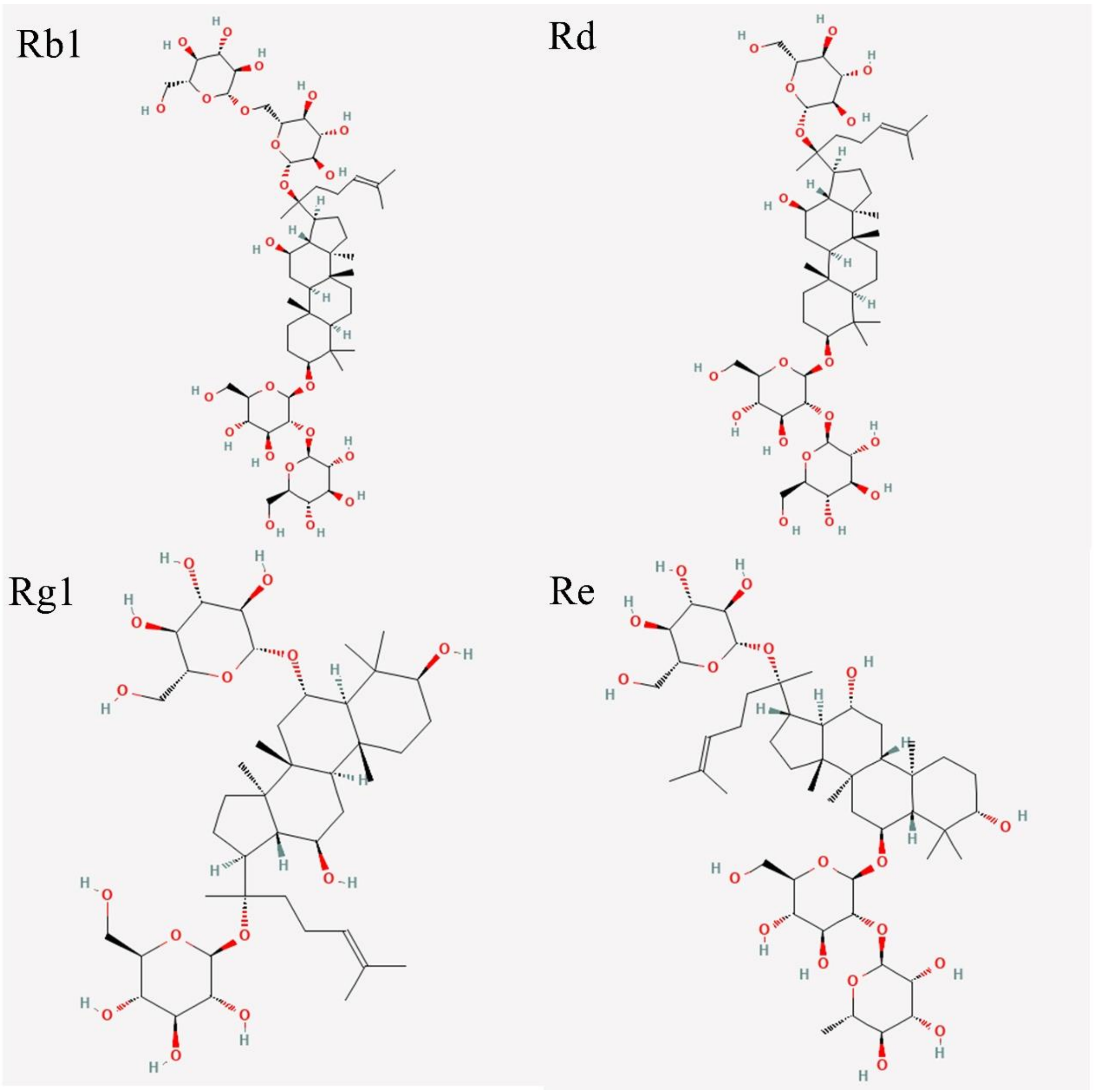

Fig. S4. The structure of ginsenoside Rg1, Re, Rb1, and Rd

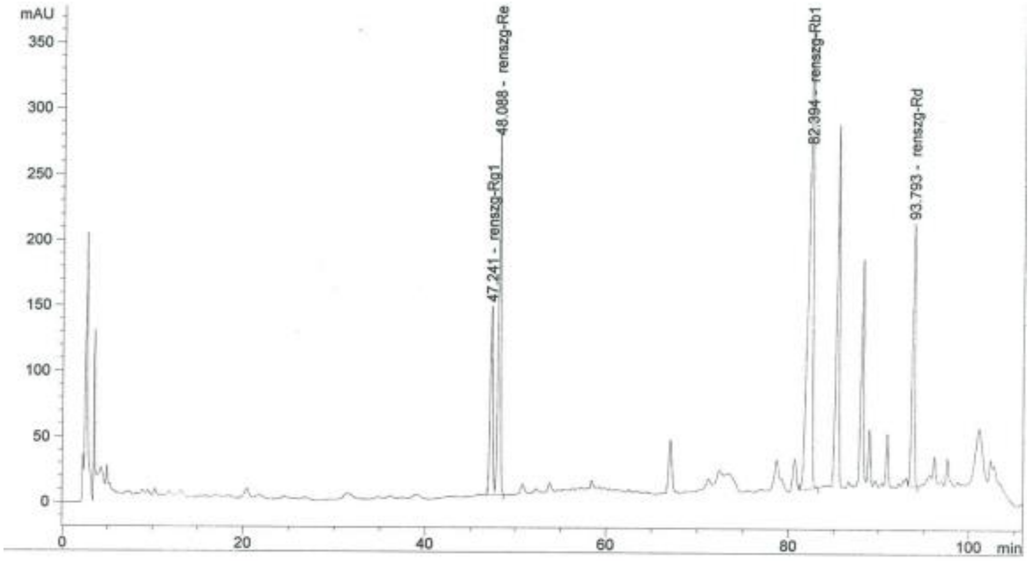

Fig. S5. HPLC fingerprint of ginseng extract

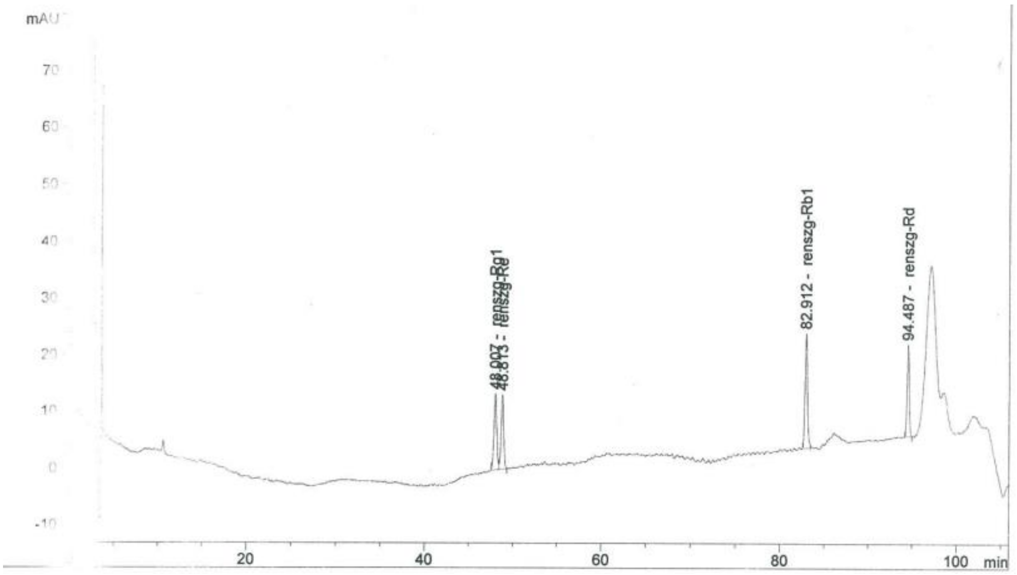

Fig. S6. HPLC fingerprint of ginsenoside Rg1, Re, Rb1, and Rd

溶液 5～20μl，注入液相色谱仪，测定，即得。

本品按干燥品计算，含人参皂苷 Rg_1（$C_{42}H_{72}O_{14}$）、人参皂苷 Re（$C_{48}H_{82}O_{18}$）和人参皂苷 Rd（$C_{48}H_{82}O_{18}$）的总量应为 30%～45%。

【贮藏】 密闭，置干燥处。

【制剂】 口服。

人参总皂苷

Renshen Zongzaogan

TOTAL GINSENOSIDE GINSENG ROOT

本品为五加科植物人参 *Panax ginseng* C. A. Mey. 的干燥根及根茎经加工制成的总皂苷。

【制法】 取人参，切成厚片，加水煎煮二次，第一次 2 小时，第二次 1.5 小时，煎液滤过，合并滤液，通过 D101 型大孔吸附树脂柱，水洗脱至无色，再用 60%乙醇洗脱，收集 60%乙醇洗脱液，滤液浓缩至相对密度为 1.06～1.08（80℃）的清膏，干燥，粉碎，即得。

【性状】 本品为黄白色或淡黄色的粉末；微臭，味苦；具吸湿性。

本品在甲醇或乙醇中易溶，在水中溶解，在乙醚或石油醚中几乎不溶。

【鉴别】 （1）取本品 0.1g，置试管中，加水 2ml，用力振摇，产生持久性泡沫。

（2）取本品 0.1g，加甲醇 10ml 使溶解，作为供试品溶液；另取人参对照药材 1g，加水 100ml 煎煮 2 小时，滤过，滤液通过 D101 型大孔吸附树脂柱（内径为 1cm，柱高为 15cm），用水洗至无色，弃去水液，再用 60%乙醇 20ml 洗脱，收集洗脱液，蒸干，残渣加甲醇 10ml 使溶解，作为对照药材溶液。再取人参皂苷 Rb_1 对照品、人参皂苷 Rg_1 对照品与人参皂苷 Re 对照品，加甲醇溶解制成每 1ml 各含 2mg 的混合溶液，作为对照品溶液。照薄层色谱法（通则 0502）试验，吸取上述三种溶液各 2μl，分别点于同一硅胶 G 薄层板上，以三氯甲烷-乙酸乙酯-甲醇-水（15：40：22：10）10℃以下放置的下层溶液为展开剂，展开，取出，晾干，喷以 10%硫酸乙醇溶液，在 105℃加热至斑点显色清晰，分别置日光和紫外光灯（365nm）下检视。供试品色谱中，在与对照药材色谱和对照品色谱相应的位置上，日光下显相同颜色的斑点，紫外光下显相同颜色的荧光斑点。

【检查】 **粒度** 依法检查（通则 0982 第二法），能通过 120 目筛的粉末不少于 95%。

干燥失重 取本品，在 105℃干燥至恒重，减失重量不得过 5.0%（通则 0831）。

总灰分 不得过 6.0%（通则 2302）。

炽灼残渣 不得过 6.0%（通则 0841）。

重金属及有害元素 照铅、镉、砷、汞、铜测定法（通则 2321）测定，铅不得过 3mg/kg；镉不得过 0.2mg/kg；砷不得过 2mg/kg；汞不得过 0.2mg/kg；铜不得过 20mg/kg。

有机氯农药残留量 照农药残留量测定法（通则 2341 第一法）测定：六六六（总 BHC）不得过 0.1mg/kg；滴滴涕（总 DDT）不得过 1mg/kg；五氯硝基苯（PCNB）不得过 0.1mg/kg。

【特征图谱】 照高效液相色谱法（通则 0512）测定。

色谱条件与系统适用性试验 以十八烷基硅烷键合硅胶为填充剂（柱长为 25cm，内径为 4.6mm，粒径为 5μm，载碳量 11%）；以乙腈为流动相 A，以 0.1%磷酸溶液为流动相 B，按下表中的规定进行梯度洗脱；柱温为 30℃；流速为每分钟 1.3ml；检测波长为 203nm。理论板数按人参皂苷 Re 峰计算应不低于 6000，按人参皂苷 Rd 峰计算应不低于 200000。

时间（分钟）	流动相 A（%）	流动相 B（%）
0～30	19	81
30～35	19→24	81→76
35～60	24→40	76→60

参照物溶液的制备 取人参皂苷 Rg_1 对照品、人参皂苷 Re 对照品和人参皂苷 Rd 对照品适量，精密称定，分别加甲醇制成每 1ml 含人参皂苷 Rg_1 0.3mg、人参皂苷 Re 0.5mg 和人参皂苷 Rd 0.2mg 的溶液，即得。

供试品溶液的制备 取本品 30mg，精密称定，置 10ml 量瓶中，加甲醇超声处理使溶解并稀释至刻度，摇匀，滤过，取续滤液，即得。

测定法 分别精密吸取参照物溶液与供试品溶液各 10μl，注入液相色谱仪，测定，即得。

供试品特征图谱中应呈现 7 个特征峰，其中 3 个峰应分别与相应的参照物峰保留时间相同；与人参皂苷 Rd 参照物峰相应的峰为 S 峰，计算特征峰 3～7 的相对保留时间，其相对保留时间应在规定值的±5%之内，规定值为：0.84（峰 3）、0.91（峰 4）、0.93（峰 5）、0.95（峰 6）、1.00（峰 7）。

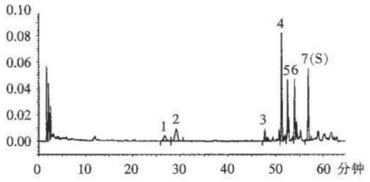

对照特征图谱

峰 1：人参皂苷 Rg_1　峰 2：人参皂苷 Re　峰 3：人参皂苷 Rf
峰 4：人参皂苷 Rb_1　峰 5：人参皂苷 Rc　峰 6：人参皂苷 Rb_2
峰 7(S)：人参皂苷 Rd

【含量测定】 **人参总皂苷** **对照品溶液的制备** 取人参皂苷 Re 对照品适量，精密称定，加甲醇制成每 1ml 含 1mg 的溶液，即得。

标准曲线的制备　精密吸取对照品溶液 20μl、40μl、80μl、120μl、160μl、200μl，分别置于具塞试管中，低温挥去溶剂，加入 1%香草醛高氯酸试液 0.5ml，置 60℃恒温水浴上充分混匀后加热 15 分钟，立即用冰水冷却 2 分钟，加入 77%硫酸溶液 5ml，摇匀；以试剂作空白。消除气泡后照紫外-可见分光光度法（通则 0401），在 540nm 的波长处测定吸光度，以吸光度为纵坐标，浓度为横坐标绘制标准曲线。

测定法　取本品约 50mg，精密称定，置 25ml 量瓶中，加甲醇适量使溶解并稀释至刻度，摇匀，精密吸取 50μl，照标准曲线的制备项下的方法，自“置于具塞试管中”起依法操作，测定吸光度，从标准曲线上读出供试品溶液中人参皂苷 Re 的量，计算结果乘以 0.84，即得。

本品按干燥品计算，含人参总皂苷以人参皂苷 Re（$C_{48}H_{82}O_{18}$）计，应为 65%～85%。

人参皂苷 Rg_1、Re、Rd　照高效液相色谱法（通则 0512）测定。

色谱条件与系统适用性试验　以十八烷基硅烷键合硅胶为填充剂；以乙腈为流动相 A，以 0.1%磷酸溶液为流动相 B，按〔特征图谱〕项表中梯度进行洗脱；检测波长为 203nm。理论板数按人参皂苷 Re 峰计算应不低于 3000。

对照品溶液的制备　取人参皂苷 Rg_1 对照品、人参皂苷 Re 对照品和人参皂苷 Rd 对照品适量，精密称定，加甲醇制成 1ml 中含人参皂苷 Rg_1 0.30mg，人参皂苷 Re 0.50mg 和人参皂苷 Rd 0.20mg 的混合溶液。

供试品溶液的制备　取〔特征图谱〕项下的供试品溶液，即得。

测定法　分别精密吸取供试品溶液 10～20μl 与对照品溶液 20μl，注入液相色谱仪，测定，即得。

本品按干燥品计算，含人参皂苷 Rg_1（$C_{42}H_{72}O_{14}$）、人参皂苷 Re（$C_{48}H_{82}O_{18}$）和人参皂苷 Rd（$C_{48}H_{82}O_{18}$）的总量计，应为 15%～25%。

【贮藏】　密闭，置干燥处。

三七三醇皂苷

Sanqi Sanchunzaogan

NOTOGINSENG TRIOL SAPONINS

本品为五加科植物三七 *Panax notoginseng*（Burk.）F. H. Chen 的干燥根及根茎经加工制成的提取物。

【制法】　取三七，粉碎成粗粉，用 60%乙醇作溶剂，浸渍 24 小时后，每千克药材以每分钟 5～8ml 进行渗漉，收集 6 倍的渗漉液，浓缩，残留物用水溶解，滤过，滤液通过 D101 型大孔吸附树脂柱，以适量水洗脱，弃去水液，再用 40%乙醇洗脱，收集洗脱液，滤过，滤液浓缩，干燥，研成细粉，即得。

【性状】　本品为浅黄棕色至黄棕色的粉末；无臭、味苦。

【鉴别】　取本品，照〔含量测定〕项下的方法试验，供试品色谱中应呈现与对照品三七皂苷 R_1、人参皂苷 Rg_1、人参皂苷 Re 色谱峰保留时间相同的色谱峰。

【检查】　**干燥失重**　取本品，以五氧化二磷为干燥剂，在室温减压干燥至恒重，减失重量不得过 7.0%（通则 0831）。

炽灼残渣　不得过 0.9%（通则 0841）。

重金属　取炽灼残渣项下遗留的残渣，依法检查（通则 0821 第二法），含重金属不得过 20mg/kg。

树脂残留　照残留溶剂测定法（通则 0861 第二法）测定。

色谱条件与系统适用性试验　以键合/交联聚乙二醇为固定相的毛细管柱（柱长为 30m，内径为 0.53mm，膜厚度为 1.0μm）；柱温为程序升温：起始温度 30℃，保持 6 分钟，再以每分钟 10℃的速率升温至 150℃，并保持 2 分钟；再以每分钟 30℃的速率升温至 180℃，保持 2 分钟。氢火焰离子化检测器检测，检测器温度 220℃，进样口温度 200℃。理论板数按苯乙烯峰计算，应不低于 20000；正己烷、苯、甲苯、苯乙烯的分离度应大于 1.5；二甲苯类峰、二乙烯苯类峰与其他峰之间的分离度应大于 1.5。

对照品贮备液的制备　取正已烷、苯、甲苯、二甲苯、苯乙烯、二乙基苯和二乙烯苯适量，精密称定，加 *N*,*N*-二甲基甲酰胺溶解并稀释成每 1ml 含正已烷、甲苯、二甲苯、苯乙烯、二乙基苯、二乙烯苯各 0.2mg 及苯 0.02mg 的混合溶液。精密量取 2.5ml，置 100ml 量瓶中，用水稀释至刻度，摇匀，作为对照品贮备液。

供试品溶液的制备　取本品 1g，精密称定，置 20ml 顶空取样瓶中，精密加入 5% *N*,*N*-二甲基甲酰胺水溶液 4ml，密封，超声处理使溶解，摇匀，在 60℃加热 50 分钟，作为供试品溶液。

对照品溶液的制备　取本品 1g，精密称定，置 20ml 顶空取样瓶中，精密加入标准贮备液 4ml，密封，超声处理使溶解，摇匀，在 60℃加热 50 分钟，作为对照品溶液。

测定法　分别精密量取对照品溶液和供试品溶液的顶空气体各 1ml，注入气相色谱仪，测定，即得。

本品含正已烷、甲苯、二甲苯、苯乙烯、二乙基苯和二乙烯苯均不得过 0.002%，苯不得过 0.0002%。

【指纹图谱】　照高效液相色谱法（通则 0512）测定。

色谱条件与系统适用性试验　以十八烷基硅烷键合硅胶为填充剂（柱长为 25cm，内径为 4.6mm，粒径为 5μm）；以乙腈为流动相 A，以水为流动相 B，按下表中的规定进行梯度洗脱；流速每分钟为 1.0ml；检测波长为 210nm。三七皂苷 R_1 与邻近色谱峰的分离度应大于 1.5，人参皂苷 Rg_1、人参皂苷 Re 色谱峰的分离度应大于 1.3。

时间（分钟）	流动相 A（%）	流动相 B（%）
0～5	15	85
5～43	15→25	85→75
43～55	25→35	75→65
55～60	35→40	65→60
60～62	40→15	60→85

参照物溶液的制备　取人参皂苷 Rg_1 对照品、人参皂苷

Fig. S7. The quality standard and extraction protocol from Chinese Pharmacopoeia

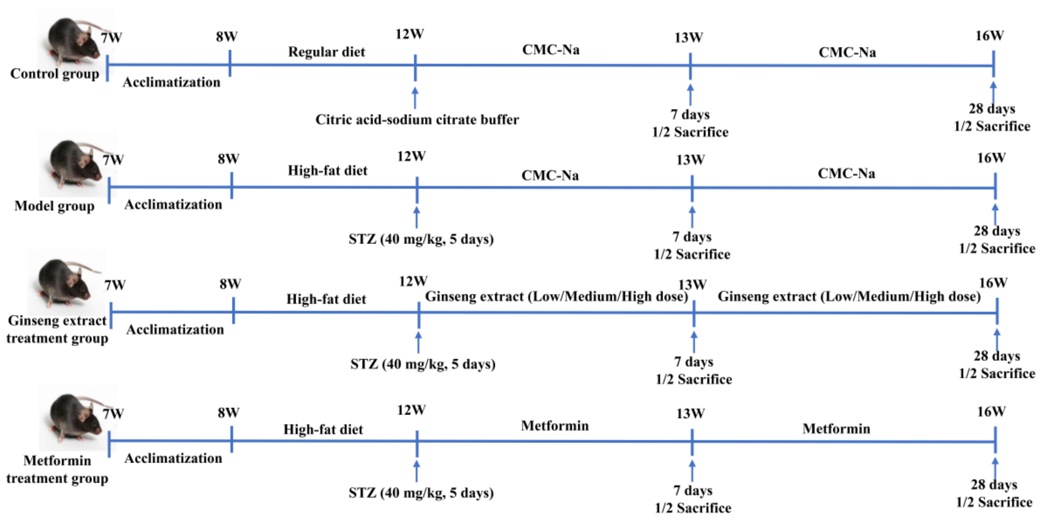

Fig. S8. Animal experiment process
